# Conditional *Dicer1* depletion using *Chrnb4-Cre* leads to cone cell death and impaired photopic vision

**DOI:** 10.1038/s41598-018-38294-9

**Published:** 2019-02-19

**Authors:** Eduardo Zabala Aldunate, Valentina Di Foggia, Fabiana Di Marco, Laura Abelleira Hervas, Joana Claudio Ribeiro, Daniel L. Holder, Aara Patel, Tommaso B. Jannini, Dorothy A. Thompson, Juan Pedro Martinez-Barbera, Rachael A. Pearson, Robin R. Ali, Jane C. Sowden

**Affiliations:** 1 Stem Cells and Regenerative Medicine Section, UCL Great Ormond Street Institute of Child Health, 30 Guilford Street, London, WC1N 1EH UK; 2 Developmental Biology of Birth Defects Section, UCL Great Ormond Street Institute of Child Health, 30 Guilford Street, London, WC1N 1EH UK; 3 UCL Institute of Ophthalmology, Department of Genetics, London, 11-43 Bath Street, London, EC1V 9EL UK; 4 Clinical and Academic Department of Ophthalmology Great Ormond Street Hospital for Children NHS Foundation Trust, London, UK

## Abstract

Irreversible photoreceptor cell death is a major cause of blindness in many retinal dystrophies. A better understanding of the molecular mechanisms underlying the progressive loss of photoreceptor cells remains therefore crucial. Abnormal expression of microRNAs (miRNAs) has been linked with the aetiology of a number of retinal dystrophies. However, their role during the degenerative process remains poorly understood. Loss of cone photoreceptors in the human macula has the greatest impact on sight as these cells provide high acuity vision. Using a *Chrnb4-cre; Dicer*^*flox/flox*^ conditional knockout mouse (Dicer CKO) to delete *Dicer1* from cone cells, we show that cone photoreceptor cells degenerate and die in the *Dicer*-deleted retina. Embryonic eye morphogenesis appeared normal in Dicer CKO mice. Cone photoreceptor abnormalities were apparent by 3 weeks of age, displaying either very short or absent outer segments. By 4 months 50% of cones were lost and cone function was impaired as assessed by electroretinography (ERG). RNAseq analysis of the Dicer CKO retina revealed altered expression of genes involved in the visual perception pathway. These data show that loss of *Dicer1* leads to early-onset cone cell degeneration and suggest that *Dicer1* is essential for cone photoreceptor survival and homeostasis.

## Introduction

Vision relies on the proper function of two types of light-sensitive photoreceptor cells: rods and cones. Although in mammals cone photoreceptors are considerably less abundant than rods, they are critical for daylight colour vision and visual acuity. Photoreceptor cells are metabolically highly active, needing high rates of protein synthesis and trafficking from the inner to the outer segments via the connecting cilium to maintain visual cycle function^1^. They are constantly under photo-oxidative stress and their lipid-enriched outer segments are vulnerable to oxidative stress. These characteristics are thought to make photoreceptors especially susceptible to degeneration^2^. While many genes have been associated with photoreceptor degeneration^1^ (RetNet http://www.sph.uth.tmc.edu/RetNet/), the molecular mechanisms leading to outer segment impairment and cell death are still poorly understood. In most conditions leading to photoreceptor degeneration, whether genetic-based or injury-induced, outer segment defects precede photoreceptor cell death^3,4^.

MicroRNAs (miRNAs) are small post-transcriptional regulators of gene expression^5,6^ shown to be important in cells that undergo cellular stress^7^. Primary miRNAs are first processed in the nucleus into precursor miRNAs by a DROSHA/DGCR8 complex and then in the cytoplasm into mature functional miRNAs by DICER1, an RNase type III endonuclease that is essential for generating mature functional miRNAs^8^. More than 250 miRNAs have been identified in the mouse neural retina^9–13^, with some fluctuating significantly in different models of photoreceptor degeneration^14,15^. For instance, the miR-183 cluster (miR-183; -182 and -96), which is the most abundant miRNA family in the retina and highly enriched in both cones and rods^9,12,16,17^ was downregulated in four models of retinitis pigmentosa^14,15^. Other studies have shown that inactivation of the miR-183 cluster results in photoreceptor degeneration upon light-induced damage^18^, or electroretinography (ERG) defects first, followed by age-induced photoreceptor degeneration^19^. Several targets of the miR-183 cluster have been recently identified, notably *Rac1*^20^, whose upregulation leads to specific rod photoreceptor degeneration^21^ and knockout promotes rod survival against photo-oxidative stress^22^. In addition, knockout of *Rncr3*, the main source of miR-124a, causes apoptosis of newly differentiated cones, together with severe CNS abnormalities^23^.

During retinal histogenesis, cone photoreceptor cells are one of five main types of retinal neuron generated from multipotential retinal progenitor cells (RPCs)^24^. Cre-mediated loss of *Dicer1* in RPCs leads to widespread ocular defects (using Chx10-, αPax6- Dkk3- and, Rx- cre-drivers), including microphthalmia, abnormal developmental timing of generation of retinal cell types, apoptosis of retinal progenitors and progressive retinal degeneration^25–28^. Less is known however, about the specific requirement for DICER1 function in individual postmitotic retinal cell types. *Dicer1* knockout (i7 Rho cre-driver) in postmitotic rods led to rod outer segment impairment by 2 months of age and loss of rods by 3.5 months of age^29^, along with downregulation of the miR-183 cluster (miR-183, miR-182, miR-96). miRNAs depletion from adult cones via *Dgcr8* knockout (D4opsin- cre-driver), led to outer segment loss by 2 months of age, accompanied by loss of cone function, but cone death was not reported^16^. Delivery of exogenous miR-183 and miR-182 stopped outer segment loss, but cone photoreceptor survival was not affected and there is some evidence that miRNAs can by-pass Drosha processing^30^.

In this study we investigated the effect of conditional *Dicer1* knockout in developing cones using a neuronal acetylcholine receptor subunit beta-4 (Chrnb4)-cre driver to elucidate directly whether DICER processing of miRNAs is needed for cone photoreceptor survival. We show that *Dicer1*-depleted cone photoreceptor cells quickly degenerate and die, thus demonstrating a role for DICER1 in cone survival. Cone ERGs were abnormal and RNAseq transcriptome analysis of the *Dicer1* CKO retina revealed gene dysregulation. These data suggest that loss of *Dicer1* function in cones leads to cone cell degeneration in a process that is reminiscent of a cone dystrophy, in which cones are primarily affected and rods remain unaffected.

## Results

### Chrnb4-cre drives recombination in developing cones

**Figure 1:**
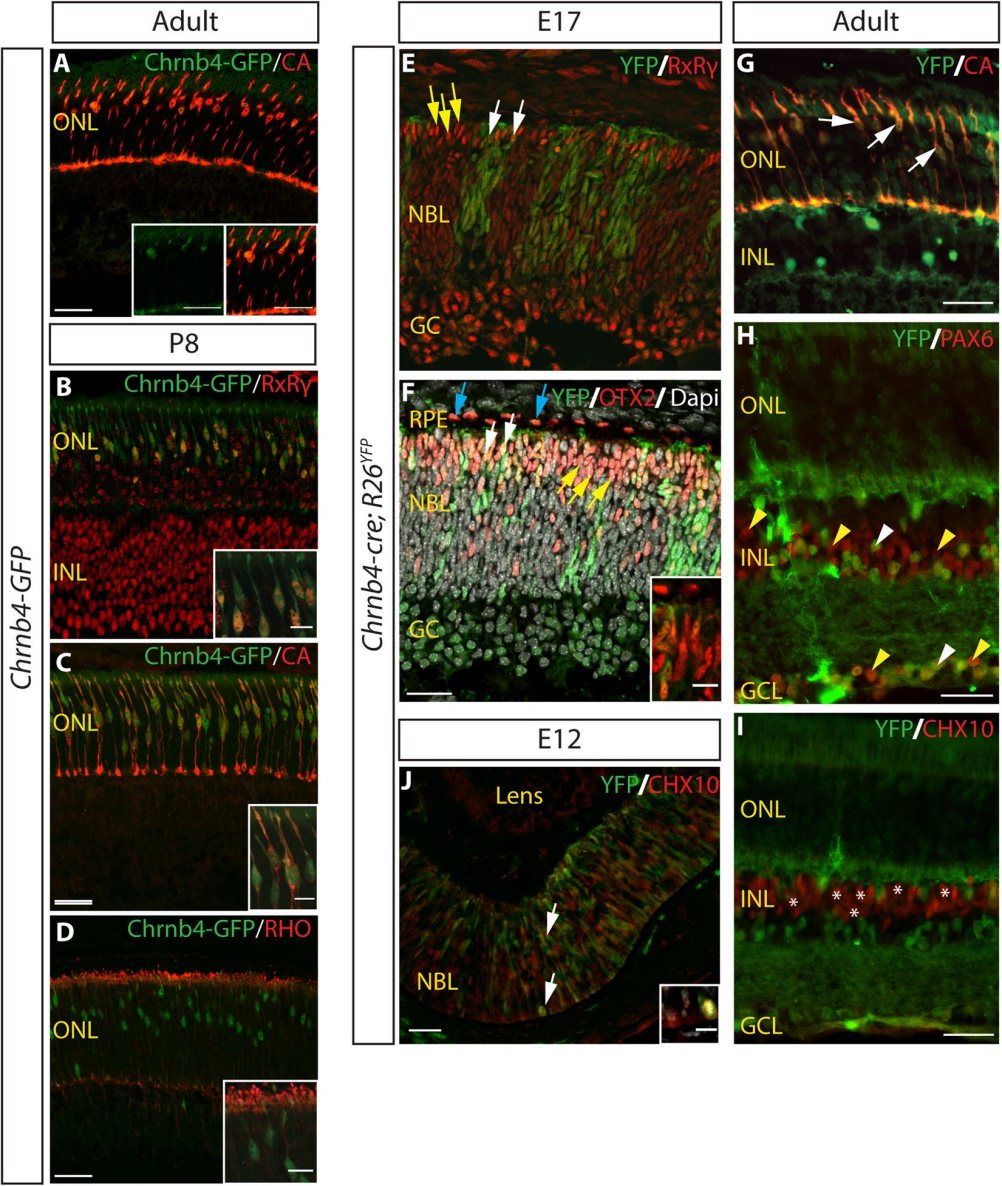
Chrnb4-GFP and Chrnb4-cre expression in developing and adult cone photoreceptor cells. **(A)** Immunostaining of adult (6 week old) *Chrnb4-GFP* retinas. Chrnb4-GFP expression co-labels with the expression of cone marker CA in the ONL. **(B–D)** Immunostaining of P8 *Chrnb4-GFP* retinas. Chrnb4-GFP expression co-labels with the expression of cone markers RxRγ **(B)** and CA **(C)** but not with the expression of rod marker RHO **(D)**. See Supplementary Figs 3 and 4 for the separate channels of the data shown in the merged images in A & B. **(E**,**F)** Immunostaining on E17 *Chrnb4-cre; R26*^*YFP*^ retinas using an anti-GFP antibody to amplify the YFP signal intensity. YFP expression is detected in some RxRγ^+ve^ cones (white arrows), while most cones remain YFP^-ve^ (**E**, yellow arrows). YFP expression is also seen in some OTX2-expressing photoreceptor progenitors (**F**, white arrows). Yellow arrows indicate OTX2^+ve^ cells that are not YFP^+ve^. OTX2^+ve^ RPE cells do not express YFP (blue arrows). **(G**–**I)** Immunostaining on adult (6 weeks old) *Chrnb4-cre; R26*^*YFP*^ retinas. YFP expression was detected in CA^+ve^ cones (**G**, white arrows) and in a few inner retinal cells including some PAX6^+ve^ cells (**H**, white arrowheads). Most Pax6^+ve^ cells in the inner retina remained YFP^-ve^ (**H**, yellow arrowheads). CHX10^+ve^ bipolar cells were mainly YFP^-ve^ (**I**, asterisks). **(J)** Immunostaining on E12 *Chrnb4-cre; R26*^*YFP*^ retinas was also performed. YFP expression was detected in a subset of CHX10^+ve^ RPCs (white arrows). CA: cone arrestin. RxRγ: retinoid x receptor gamma. RHO: rhodopsin. RPE: retinal pigment epithelium. ONL: outer nuclear layer. INL: inner nuclear layer. GCL: ganglion cell layer. RPC: retinal progenitor cells. Scale bars: 30 μm. Scale bars of insets: 10 μm.

Using *Chrnb4-GFP* BAC transgenic mice^31^, we confirmed the previously reported expression of the Chrnb4-GFP transgene specifically in cone photoreceptors of the adult retina^32^ (Fig. 1A). Chrnb4-GFP expression co-labelled with cone markers RxRγ and cone arrestin (CA) (Fig. 1B,C) by postnatal day P8, indicating that Chrnb4-GFP is also a marker of postnatal developing cones (Fig. 1). A recent paper also reported expression in a sub-population of early retinal progenitors that is progressively restricted to maturing cones^33^. Together these data indicate that a Chrnb4-cre driver may be useful for cone conditional ablation studies. Next, we crossed a Chrnb4-cre BAC transgenic mouse line generated using the same BAC clone as *Chrnb4-GFP* mice^31^ with *R26*^*YFP*^ mice^34^ in order to assess the recombination profile of the Cre recombinase driven by the Chrnb4 promoter. By E17 in *Chrnb4-cre; R26*^*YFP*^ retinas, YFP expression was detected in a few RxRg-positive (Fig. 1E, white arrows) and OTX2-positive cells (Fig. 1F, white arrows), markers for cone precursor and photoreceptor progenitor cells respectively. Some patchy distribution of YFP was also observed extending across the full thickness of the retina indicative of clonal descendants of single recombined progenitors (Fig. 1E,F). By 6 weeks of age, Chrnb4-cre-mediated recombination had occurred in most cone arrestin-expressing cones (Fig. 1G, white arrows) consistent with Chrnb4-cre activity in cone photoreceptor cells. YFP expression was also detected in a number of Pax6-expressing inner retinal cells (Fig. 1H, white arrow heads), but was not detected in CHX10-positive bipolar cells (Fig. 1I, asterisks). Analysis of earlier embryonic stages showed that at E12, when the retina is mainly comprised of retinal progenitor cells (RPCs), a small subset of CHX10-positive, YFP-positive RPCs was observed (Fig. 1J, white arrows) with a variable distribution between embryos, suggesting that the YFP positive inner retinal cells observed in adult mice derive from these RPCs, as Chrnb4GFP expression was not detected in adult INL cells (Fig. 1A). No YFP expression was detected in RPE cells (Fig. 1F, blue arrows). This analysis demonstrated that Chrnb4-cre drives recombination mostly in cones, with additional Cre activity in some progenitor cells leading to labelled progeny cells in the INL and GCL.

### Normal retinal histogenesis and cone birthing in Dicer CKO

**Figure 2:**
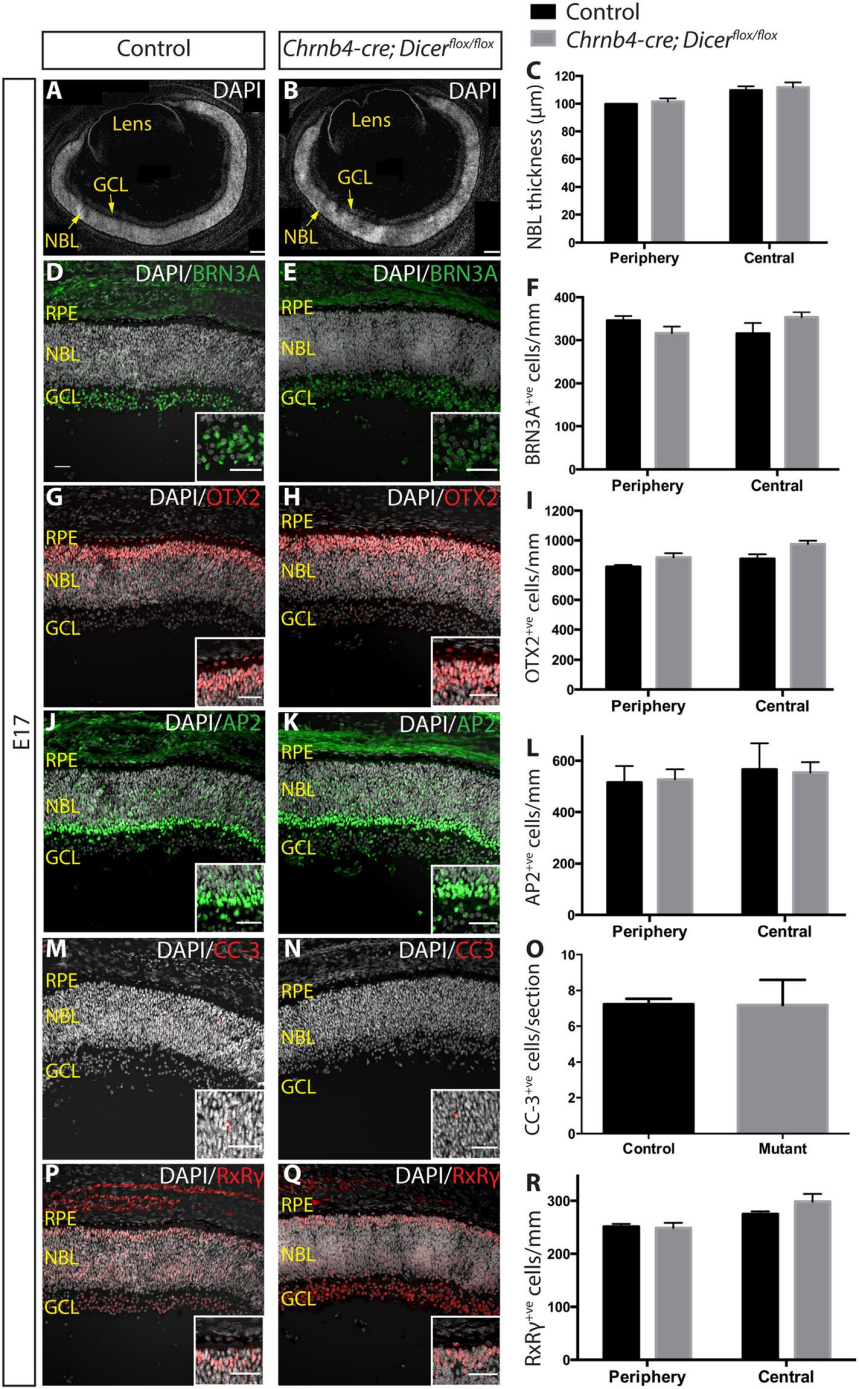
Retinal histogenesis and cone birthing not affected in *Chrnb4-cre; Dicer*^*flox/flox*^ mice at E17. **(A**,**B)** Reconstruction of E17 control **(A)** and Dicer CKO **(B)** retinas. **(C)** Measurement of the NBL. Immunohistochemistry and quantification of cells were carried out for BRN3A^+ve^ ganglion cells **(D**–**F)**, OTX2^+ve^ photoreceptor progenitors **(G**–**I)**, AP2^+ve^ amacrine and horizontal cells **(J–L)**, CC-3^+ve^ apoptotic cells **(M**–**O)** and RxRγ^+ve^ cone precursors **(P**–**R)** in control and Dicer CKO E17 retinas. See Supplementary Figs 5–8 for the separate channels of the data shown in the merged images. Error bars indicate standard error of the mean. NBL: neuroblastic layer. GCL: ganglion cell layer. RPE: retinal pigment epithelium. CKO: conditional knockout (*Chrnb4-cre; Dicer*^*flox/flox*^). Controls: *Dicer*^*flox/+*^ and *Dicer*^*flox/flox*^ littermates of Dicer CKO. Scale bars of A and B: 100 μm. Scale bars of D-Q: 30 μm. Scale bars of insets: 10 μm.

To assess the *in vivo* physiological impact of the conditional loss of *Dicer* using the Chrnb4-cre driver, crosses with *Dicer*^*flox/flox*^ knock-in mice were made to generate *Chrnb4-cre; Dicer*^*flox/flox*^ conditional knockout mice (referred to as Dicer CKO). Recombination of the Dicer locus in Dicer CKO retina was confirmed by genomic PCR and Reverse transcription (RT) PCR; deletion of the region encoding the essential Dicer RNaseIII domain (exons 20 and 21) was detected in retinal DNA and Dicer mRNA (Supplementary Fig. 1). By E17 the eye morphology of Dicer CKO was not different from control mice and no signs of microphthalmia were detected (Fig. 2A,B). Like in control mice, the forming retinal ganglion cell layer was visible (Fig. 2A,B) and the neuroblastic layer (NBL) displayed no significant thickness difference (Fig. 2C), suggesting Dicer CKO mice did not display embryonic apoptotic, or proliferative defects, despite the low level of Cre activity in RPCs.

As depletion of *Dicer* in Pax6-expressing RPCs was previously reported to cause an increased number of peripheral early born cell types by E16^26^, we carried out an analysis of the NBL to assess whether low levels of RPC recombination by E17 could have led to a similar early phenotype. No significant difference was observed in the number of peripheral or central BRN3A^+ve^ ganglion cells (Fig. 2D–F), OTX2-expressing photoreceptor progenitors (Fig. 2G–I), and AP2^+ve^ horizontal and amacrine cells (Fig. 2J–L) between Dicer CKO and control mice. Moreover, no significant difference was detected in the number of cleaved caspase 3-expressing cells (Fig. 2M–O). These results show that, in line with earlier studies^25^ (using *Chx10-Cre; Dicer*^*flox/flox*^ mice), mosaic recombination observed in a few RPCs did not lead to any early major morphological defect.

To assess if cone birthing was affected, we counted the number of peripheral and central RxRg^+ve^ cone precursors^35^ and found no significant differences between control and Dicer CKO retinas (Fig. 2P–R). Considering that under normal conditions all cones in the central retina and most cones in the peripheral retina are already born by E17^36^, these data suggest that cone photoreceptor precursor cell genesis is not perturbed in Dicer CKO mice. Together these data indicate that Dicer CKO mice display normal early eye formation and early retinal development and retinogenesis, with all cone precursor cells being born.

### Cone and inner retinal abnormalities by P21 in Dicer CKO

**Figure 3:**
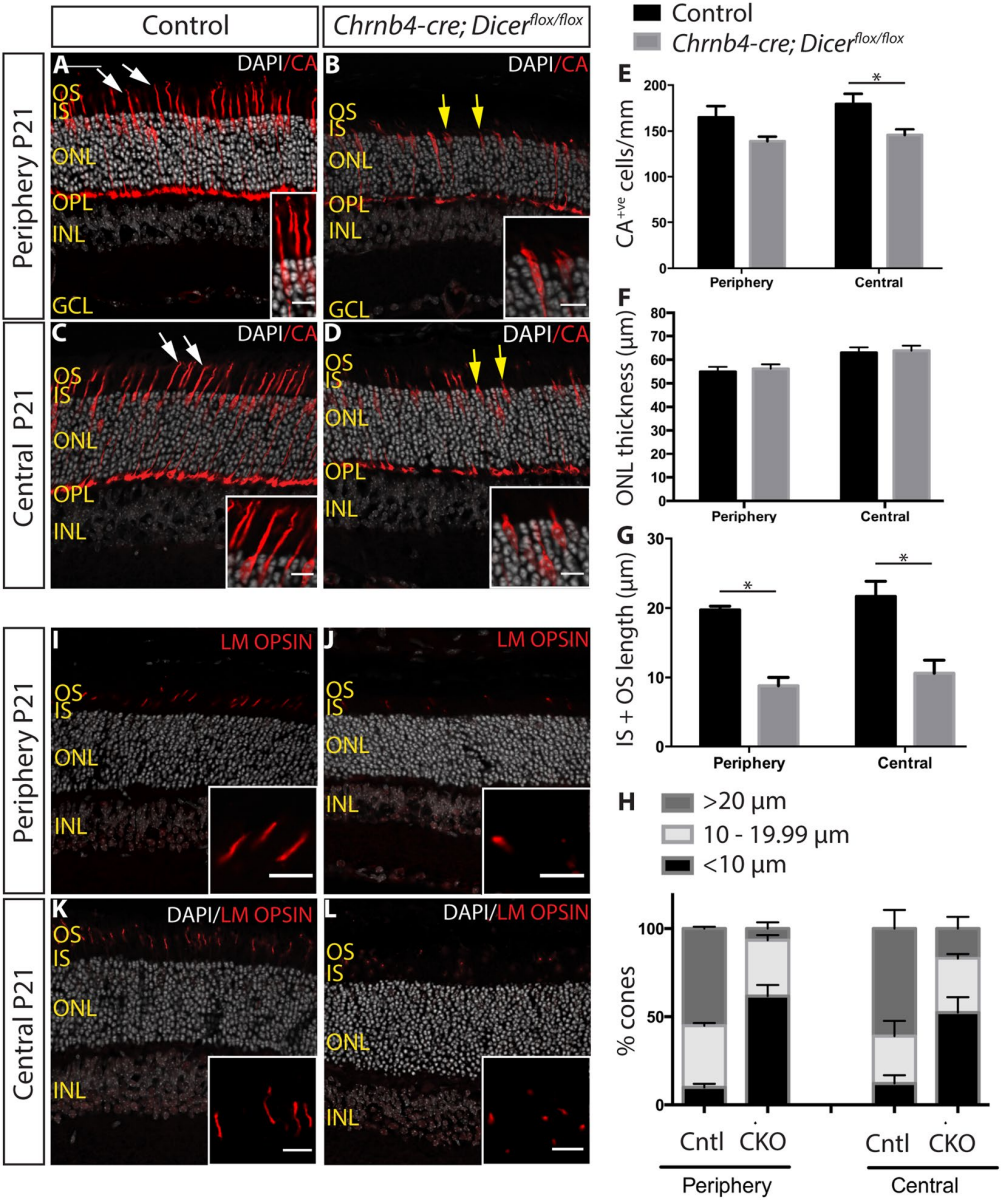
Cone abnormalities by P21 in Dicer CKO mice. **(A**–**D)** Immunostaining for CA of P21 control **(A**,**C)** and Dicer CKO **(B**,**D)** peripheral and central retinas. White arrows indicate normal wild type segments. Yellow arrows indicate abnormal short segments **(E)** Quantification of CA^+ve^ cone photoreceptors. **(F)** Measurement of the ONL thickness. **(G)** Average length of CA^+ve^ segments (IS + OS). **(H)** Graph showing the percentage of cones displaying long (>20 μm), medium (10–19.99 μm) and short segments (<10 μm) in control and Dicer CKO peripheral and central retinas. **(I**–**L)** Immunostaining for the cone outer segment marker LM OPSIN. OS: outer segments. IS: inner segments. ONL: outer nuclear layer. INL: inner nuclear layer. OPL: outer plexiform layer. GCL: ganglion cell layer. CA: cone arrestin. *Indicates p-value < 0.05. Mann-Whitney non parametric test was used. Error bars indicate standard error of the mean. Scale bar: 30 μm. Scale bar of insets: 10 μm.

By postnatal day (P) 21, wild type cone photoreceptors display inner and outer segments; both cell bodies and segments were immuno-labelled by cone arrestin (CA) (Fig. 3A,C, white arrows). In Dicer CKO mice impaired or absent cone inner/outer segments were readily apparent (Fig. 3B,D, yellow arrows) compared to controls. Counting of CA^+ve^ cones showed a 15.7% and an 18.8% decrease in the number of cones in Dicer CKO peripheral (165.1 ± 12.2 CA^+ve^ cells/mm in control and 139.1 ± 5.1 CA^+ve^ cells/mm in Dicer CKO; p = 0.1508) and central retinas (179.6 ± 10.9 CA^+ve^ cells/mm in control and 145.9 ± 6.0 CA^+ve^ cells/mm in mutant; p = 0.0317), respectively (Fig. 3E). Conversely, when the thickness of the ONL was measured no apparent size difference was observed, suggesting that rod survival was not significantly affected (Fig. 3F). The CA staining also revealed the strikingly shortened segments of the remaining cones (Fig. 3A–D, yellow arrows) and the abnormal outer segments in Dicer1 CKO mice were confirmed by LM opsin staining (Fig. 3I-L). Dicer-depleted cones displayed much shorter segments on average (19.7 ± 0.5 μm vs 8.8 ± 1.2 μm in the periphery, p = 0.0286 and 21.7 ± 2.2 μm vs 10.6 ± 1.9 μm in the central retina, p = 0.0286) (Fig. 3G); with over 50% of cones (61.6% in the periphery vs 52% in the central retina) displaying very short or absent segments (<10 μm), compared to less than 12% in the control retinas (9.8% in the periphery vs 12% in the central retina) (Fig. 3H).

**Figure 4:**
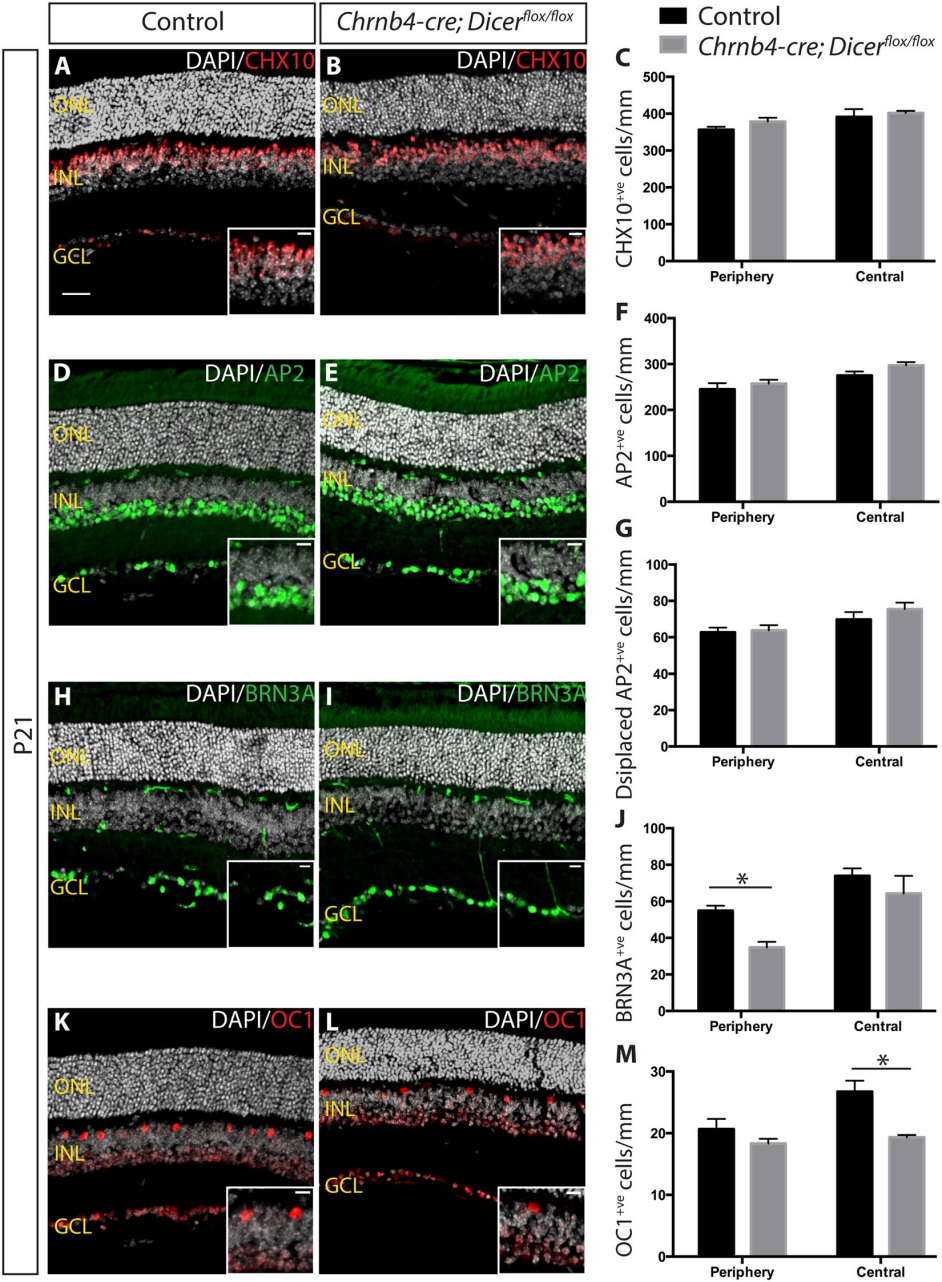
Abnormalities in some inner retinal cells by P21 in Dicer CKO. Immunostaining and quantification of CHX10^+ve^ bipolar cells **(A**–**C)**, AP2^+ve^ amacrine and displaced amacrine cells **(D**–**G)**, BRN3A^+ve^ ganglion cells **(H**–**J)**, and OC1^+ve^ horizontal cells **(K**–**M)**, for control and Dicer CKO peripheral and central retinas. See Supplementary Figs 9–11 for the separate channels of the data shown in the merged images in A, B, H, I, K, L. ONL: outer nuclear layer. INL: inner nuclear layer. GCL: ganglion cell layer *indicates p-value < 0.05. Mann-Whitney non parametric test was used. Error bars indicate standard error of the mean. Scale bar: 30 μm. Scale bar of insets: 10 μm.

By contrast to the observed effect on cone cells, INL thickness was not significantly different between control and Dicer CKO retinas (Fig. 4 and data not shown). Counting CHX10^+ve^ bipolar (Fig. 4A–C) and AP2^+ve^ amacrine cells (Fig. 4D–G) revealed no significant differences. However, BRN3A-expressing ganglion cells (Fig. 4H–J) and OC1-expressing horizontal cells (Fig. 4K–M) were partially depleted in Dicer CKO retinas, consistent with the *Chrnb4-cre; R26*^*YFP*^ expression observed in a subset of these cells (Fig. 1H). The number of BRN3A^+ve^ ganglion cells was significantly decreased, by 37%, in the periphery of Dicer CKO retinas when compared to controls (34.8 ± 3.1 cells/mm in Dicer CKO vs 54.9 ± 2.8 cells/mm in controls, p = 0.0286), whereas the effect in the central retina was non-significant (13% reduction; 64.3 ± 9.7 cells/mm in Dicer CKO vs 73.9 ± 4.1 cells/mm in controls, p = 0.4857) (Fig. 4J). Conversely, a significant 28% decrease in the number of central OC1-expressing horizontal cells was observed in Dicer CKO (19.3 ± 0.37 cells/mm, p = 0.0286) when compared to control retinas (26.7 ± 1.8 cells/mm) but differences were not observed in the peripheral retina (18.3 ± 0.78 cells/mm in Dicer mice vs 20.7 ± 1.7 cells/mm in controls, p = 0.6571) (Fig. 4M). These results suggest loss of Dicer negatively affected the maturation and/or survival of ganglion and horizontal cells, in addition to cone cells. As neither horizontal cell ablation in mice^37^ nor ganglion cells loss in monkey eyes led to cone cell death^38^, it appears unlikely that the loss of horizontal and ganglion cells observed in Dicer CKO retina led to cone cell loss and suggests that the cone loss was cell autonomous.

### Cone photoreceptor degeneration

**Figure 5:**
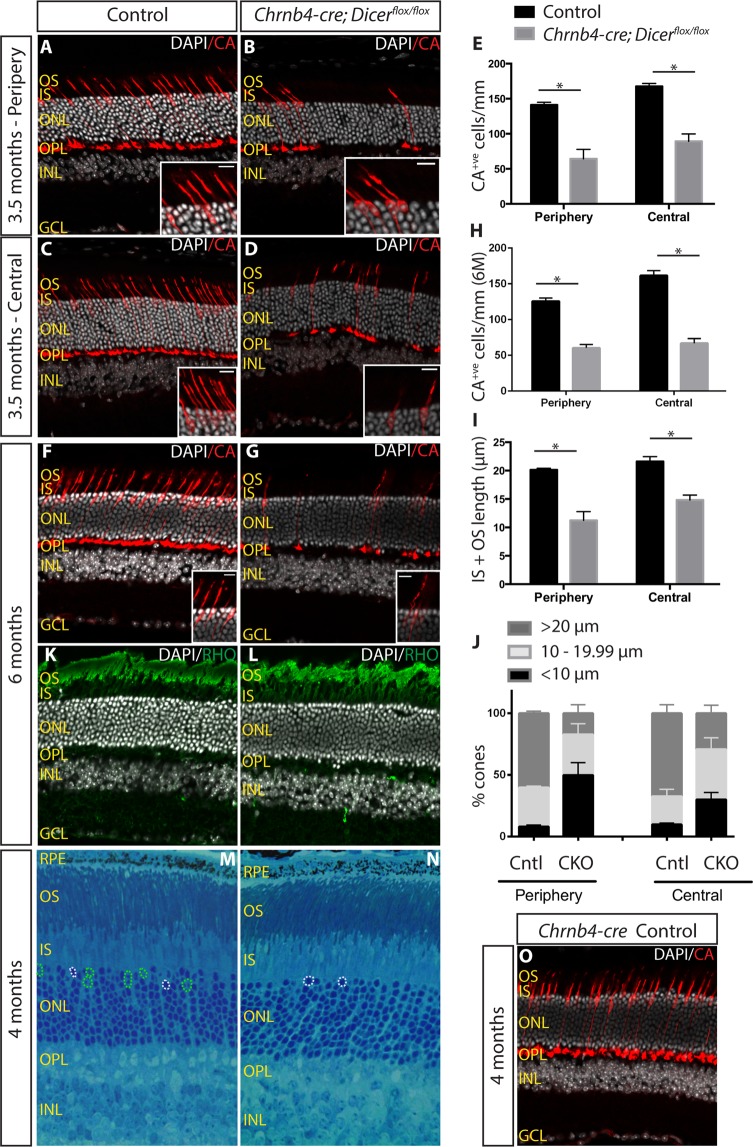
Progressive cone photoreceptor degeneration. **(A**–**D)** Immunostaining with CA of 3.5 month old control **(A**,**C)** and Dicer CKO **(B**,**D)** peripheral and central retinas. **(E)** Quantification of CA^+ve^ cone photoreceptors in 3.5 month old retinas. **(F**–**G)** Immunostaining for CA of 6 month old control **(F)** and Dicer CKO **(G)** retinas. **(H)** Quantification of CA^+ve^ cone photoreceptors in 6 month old retinas. **(I)** Average length of CA^+ve^ segments (IS + OS) in 3.5 month old retinas. **(J)** Graph showing the percentage of cones displaying long (>20 μm), medium (10–19.99 μm) and short segments (<10 μm) in control and Dicer CKO peripheral and central 3.5 month old retinas. **(M**,**N)** Toluidine blue staining of 4 month old control and Dicer CKO retinas. Green dashed circle indicate cone nuclei. White dashed circles indicate rod nuclei. **(O)** CA staining on *Chrnb4-cre* 4 month old retinas. OS: outer segments. IS: inner segments. ONL: outer nuclear layer. INL: inner nuclear layer. OPL: outer plexiform layer. GCL: ganglion cell layer. CA: cone arrestin. *Indicates p-value < 0.05. Mann-Whitney non parametric test was used. Error bars indicate standard error of the mean. Scale bar: 30 μm. Scale bar of insets: 10 μm.

To address whether cone cells exhibiting impaired segment structure by P21 showed progressive degeneration, we analysed the Dicer CKO retinas at both 3.5 and 6 months of age. Immunohistochemical analysis for cone arrestin (Fig. 5) revealed a further significant severe reduction in the number of cones by 3.5 months in both the peripheral (54.4% decrease: 141.1 ± 3.8 CA^+ve^ cones/mm in control vs 64.3 ± 13.4 CA^+ve^ cones/mm in CKO, p = 0.0286) and central Dicer CKO retinas (46.74% decrease: 167.6 ± 4.1 CA^+ve^ cones/mm in control vs 89.3 ± 10.5 CA^+ve^ cones/mm in CKO, p = 0.0286) (Fig. 5A–E). Analysis of 6 month old Dicer CKO retinas also displayed a significant reduction in cone cell numbers compared to age-matched controls (52.7% decrease in the periphery: 125.8 ± 4.5 CA^+ve^ cones/mm in control vs 60.3 ± 5.5 CA^+ve^ cones/mm in CKO and 58.5% decrease in the central retina: 161.6 ± 7.0 CA^+ve^ cones/mm in control vs 67.0 ± 7.6 CA^+ve^ cones/mm in CKO, p < 0.05) with the level of cell loss at this time point similar to that at 3.5 months (Fig. 5F–H). Like at P21, the segments of the remaining cone photoreceptors at 3.5 months of age were shorter on average in Dicer CKO in both the periphery (20.1 ± 0.3 μm in control and 11.2 ± 1.6 μm in CKO, p = 0.0286) and central retinas (21.6 ± 0.8 μm in control and 14.8 ± 0.9 μm in CKO, p = 0.0286), with most of the remaining cones having very short or absent segments (Fig. 5I,J).

The thickness of the ONL remained unchanged compared to control, indicating rod survival was not affected (Fig. 5A–L). As Muller glial cell GFAP reactivity is a common indicator of glial hypertrophy and activation in retinal degeneration we compared GFAP immuno-staining between the control and Dicer CKO retina. Some increased GFAP reactivity was apparent in the Dicer CKO retina, while laminar retinal architecture was maintained (Supplementary Fig. 2). In addition, toluidine blue staining on 4 month old retinas revealed no differences in the length and orientation of rod segments between control and Dicer CKO mice (Fig. 5M,N). Furthermore, rhodopsin staining was still visible in 6 month Dicer knockout retinas (Fig. 5K,L). Together these data indicate that loss of cone photoreceptors did not affect rod survival and morphology. As toluidine blue staining distinguishes the nuclear morphology of cone photoreceptors based on their heterochromatin (Fig. 5M,N, green dashed lines), we found that control retinas could be readily differentiated from mutant retinas (less cones) (Fig. 5M,N), further demonstrating the cone survival defects observed by immunohistochemistry. The morphology of the INL appeared normal by 3.5 months (Fig. 5M,N) and no difference in its thickness was observed between control and Dicer CKO retinas (data not shown). In addition, 3.5 month old *Chrnb4-cre* control retinas displayed normal cone morphology (Fig. 5O), indicating the cre recombinase did not have cytotoxic effects.

### Impaired retinal function in Dicer CKO

**Figure 6:**
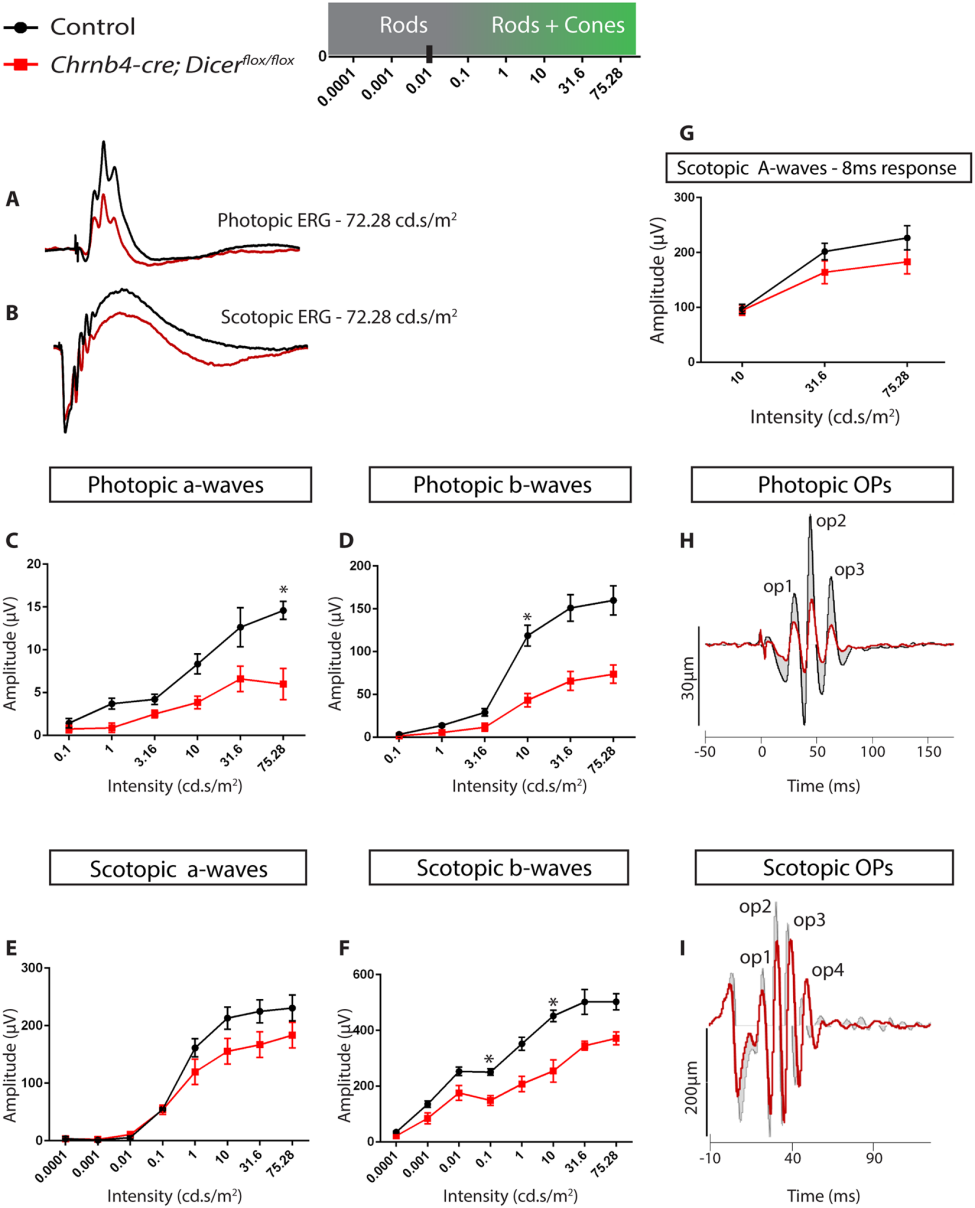
Impaired retinal function in Dicer CKO. **(A)** Photopic waveforms of 4 month old control (black) and Dicer CKO (red) retinas at 72.25 cd.s/m^2^. **(B)** Scotopic waveforms of 4 month old control (black) and Dicer CKO (red) retinas at 72.25 cd.s/m^2^. **(C,D)** Light adapted photopic a- and b-wave amplitudes plotted at different light intensities for both control and Dicer CKO retinas. **(E**,**F)** Dark adapted scotopic a- and b-wave amplitudes plotted at different light intensities for both control and Dicer CKO retinas. **(G)** Scotopic 8 ms a-wave amplitudes for control and Dicer CKO retinas. **(H)** Fourrier analysis of photopic OPs at 72.25 cd.s/m^2^. **(I)** Fourrier analysis of scotopic OPs at 72.25 cd.s/m^2^. Cd: candela. OPs: Oscillatory potentials. Multiple T-test was performed. Sidak-Bonferroni was applied to correct for multiple testing. Adjusted p-value < 0.05 (*) was considered as significant. Error bars indicate standard error of the mean.

Electroretinography (ERGs) was performed to assess the effect of *Dicer1* loss on retinal function in Dicer CKO mice. In line with the observed 50% loss of cones, photopic a-wave amplitudes, which reflect photoreceptor function, were significantly reduced in 4 month Dicer CKO when compared to controls (Fig. 6A,C). a-wave peak times were not significantly different between the two groups (data not shown). The photopic b-wave amplitudes, which depend on the presence of the a-wave and reflect photoreceptor function, synaptic transmission from photoreceptors to bipolar cells and bipolar cell function^39^ were also reduced (Fig. 6A,D). These data indicate the overall function of cone photoreceptors within the Dicer CKO retina was impaired when compared to control. Conversely, scotopic a-wave amplitudes were not significantly affected, indicating that rod function was not significantly affected (Fig. 6B,E). A trend of reduced scotopic a-wave amplitudes was however observed at high flashes (10, 31.6 and 75.28 cd.s/m^2^) (Fig. 6E), which could be due to the mixed cone/rod response obtained at these higher light intensities^40^. To isolate the rod component of the a-wave at these intensities, the scotopic a-wave amplitudes at 8 ms were plotted at 10, 31.6 and 75.25 cd.s/m^2^ (Fig. 6G). Again, no significant difference was observed between control and Dicer CKO retinas, indicative of normal rod function and consistent with our histochemical observations. Scotopic b-waves were however significantly reduced in Dicer CKO retinas (Fig. 6F), which may be attributable to the impaired cone function, or could possibly reflect the horizontal cell loss in the Dicer CKO retina, as has been previously described in a study ablating horizontal cells^37^.

We also examined the oscillatory potentials (OPs), which are dependent upon spiking neurons, amacrine and ganglion cells in the inner retina (Fig. 6H,I). The Fourier power of photopic OPs centred around 57 Hz, compared to 107 Hz for the scotopic OPs. In the time domain the individual OP of highest amplitude (op2) produced by the strongest flash was 11 ms slower photopically, than scotopically. There was, however, no difference in time to peak of rod, or cone, OPs between wild type and Dicer CKO extracted with 40–300 Hz or 60–300 Hz (Fig. 6I,H); mean values fell within +/− 2 ms. By contrast, cone, but not rod, OP amplitudes from Dicer CKO were smaller than controls (Fig. 6H). This was noted especially to the higher flash strengths.

### Transcriptome analysis of Dicer CKO shows impairment of visual perception gene expression

**Figure 7:**
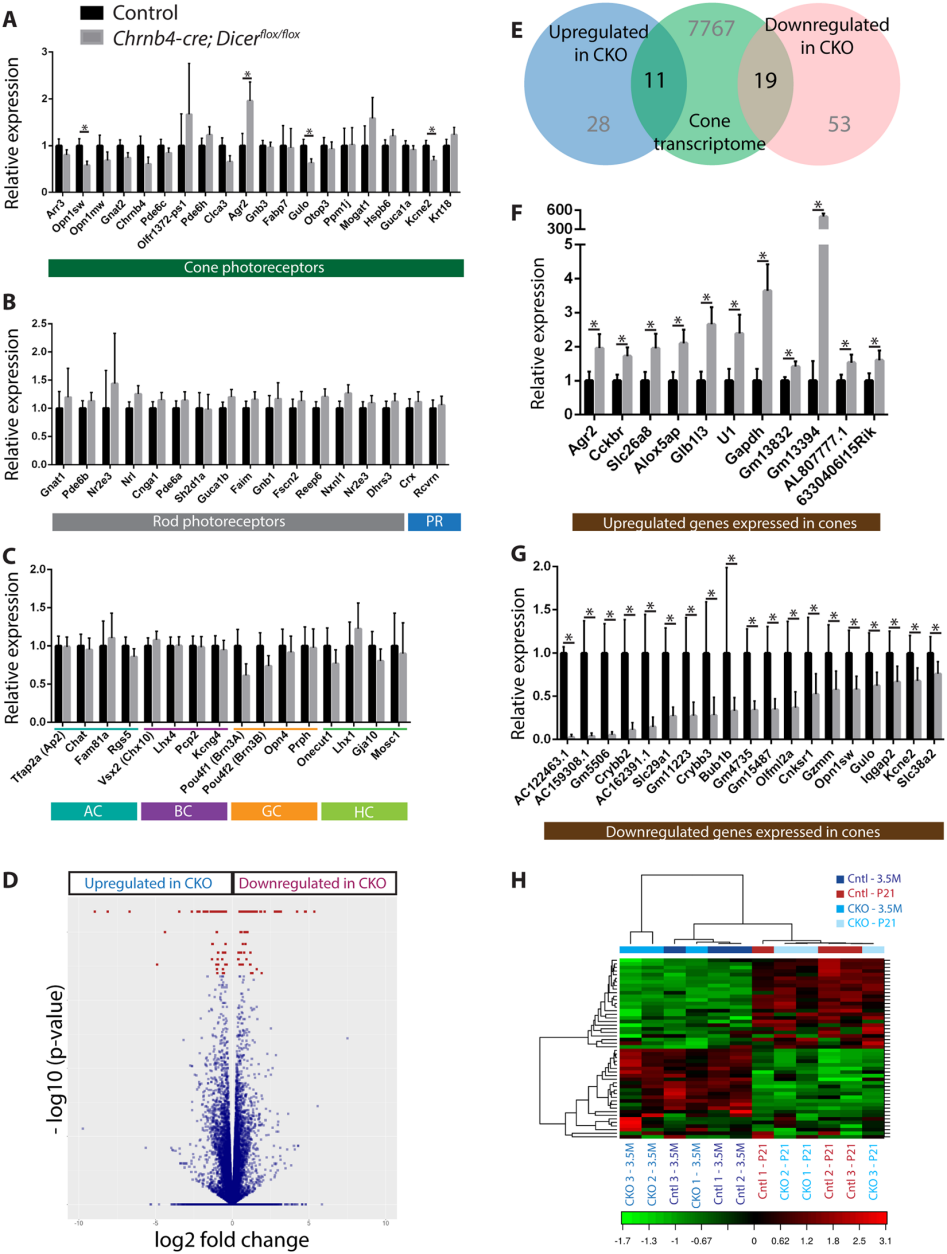
Transcriptome analysis of control and Dicer CKO P21 retinas. **(A**–**C)** RNAseq analysis of control and Dicer CKO P21 retinas. Relative expression of a set of cone-enriched **(A)**, rod-enriched **(B)**, amacrine cell, bipolar cell, ganglion cell and horizontal cell-enriched **(C)** genes normalised to the control, using FPKM values. **(D)** Volcano plot displaying the differentially expressed genes between control and Dicer CKO retinas at P21. In red, 111 genes were significantly differentially expressed, 72 downregulated and 39 upregulated in Dicer CKO retinas. **(E)** Relative expression 11 upregulated **(F)** and 19 downregulated **(G)** genes expressed in cones, normalised to the control using FPKM values **(H)** Heatmap of a two-way hierarchical clustering based on 50 known miRNAs that displayed the highest coefficient of variation across all 12 samples. Each row represents a miRNA and each column represents a sample. The colour indicates the relative expression level of the given miRNA; with red representing an expression above the mean and green below the mean. Cntl: control. CKO: conditional knockout (Dicer CKO mice Cuffdiff was used to identify significantly differentially expressed genes from the entire RNAseq dataset. p-values were adjusted using the Benjamini-Hochberg False Discovery Rate (FDR) approach to correct for multiple testing. Corrected p-value < 0.05 (*) was considered as significant. Error bars indicate standard error of the mean. PR: photoreceptors.

To determine which gene networks and pathways are affected upon *Dicer* depletion that lead to a progressive early onset cone degeneration and retinal dysfunction, the whole retinal transcriptome of Dicer CKO mice was compared to controls at P21 (when an initial small reduction in cone cell numbers was observed). We first investigated whether ablation of *Dicer* directly affected levels of known markers of cone cells (Fig. 7A) and other specific cell types (Fig. 7B,C). By plotting the expression levels of a set of genes previously reported to be enriched in cones^41,42^, we observed that the majority of these genes were downregulated in Dicer CKO when compared to controls, with *Opn1sw* (S-opsin) (fold change downregulation of 1.73, adjusted p < 0.0085), *Gulo* (fold change downregulation of 1.61, adjusted p < 0.0085) and *Kcne2* (fold change downregulation of 1.47, adjusted p < 0.0085) being significant (Fig. 7A). These reductions in cone gene expression were larger than would be expected from a 17% reduction in cone cell numbers at P21 and suggest dysregulation of cone transcripts. By contrast, *Agr2* was significantly upregulated by almost two fold in Dicer CKO retinas (adjusted p = 0.0470) and three other cone transcripts also displayed a pattern of upregulation (*Olfr1372-ps1*, *Mogat1*, *Krt18*) (Fig. 7A), indicating these genes could be direct targets of miRNAs that are depleted by loss of Dicer function.

We also examined levels of rod-enriched transcripts (Fig. 7B) as well as transcripts for amacrine, bipolar, ganglion and horizontal cell markers^41,42^ (Fig. 7C). Contrary to cone markers, rod transcripts were not significantly different between control and mutant and, in most cases, displayed a slight pattern of upregulation (Fig. 7B). Furthermore, pan photoreceptor markers *Crx* and *Rcvrn* (Recoverin) were not significantly different (Fig. 7B). Together these data suggest that *Dicer* depletion affected the cone transcriptome and not the rod transcriptome, consistent with our previous observations. There were no differences between Dicer CKO and control retinas when comparing known bipolar and amacrine cell markers (Fig. 7C), consistent with the survival of these cell types. On the other hand, *Pou4f1* (Brn3A) showed a non-significant 38.5% decrease, concomitant with the 25.5% decrease in immuno-positive cells in the whole retina (Fig. 7C). Likewise, *Onecut1* transcript displayed a 23% decrease (Fig. 7C).

Next, we performed an unbiased analysis of the RNAseq data. In total, 111 genes were significantly differentially expressed between control and Dicer CKO (Fig. 7D). Among these, 72 were downregulated in Dicer CKO (Supplementary Table 1), while 39 were upregulated (Supplementary Table 2). We checked which of these genes were normally expressed in cones by cross referencing with cone transcriptome data generated from RNAseq analysis of FACS-isolated wild type P8 and P42 Chrnb4-GFP^+ve^ cells (Fig. 7E). 11 of the upregulated genes (Fig. 7F) and 19 of the downregulated genes (Fig. 7G) were expressed in wild type cone cells from RNAseq analysis, including several that are uncharacterised (Fig. 7F,G).

Gene ontology enrichment analysis of the 111 differentially expressed genes (Supplementarys Tables 3 and 4) showed sensory perception of light stimulus (combined score of 37.7) being the top biological process term (Supplementary Table 3) and structural constituent of eye lens (combined score 95.8) being the top molecular function term (Supplementary Table 3), respectively. More than half (56 genes) had a fold change difference higher than 2 (log2 FC < 1). Notable genes that were significantly downregulated in the Dicer CKO retinas were: *Dio2* (fold change of 1.59, adjusted p < 0.0085), which is needed for the catalysis of T4 into T3 and its inhibition has been recently linked with promoting cone survival^43^; *Ptgds*, prostaglandin-D2 (fold change of 1.69), which is a ciliary protein that interacts with TOPORS; mutations in the latter being associated with retinitis pigmentosa^44^. Other dysregulated genes were enriched for GO terms involved with the extracellular matrix organisation and structure (*Col18a1*, *Ttr*, *Abi3bp*, *Col8a2*, *Itgb8*, *Col8a1*, *Fbln1*, *Fmod*, *Olfml2a*, *Thbs1*, combined score of 18.4) (Supplementary Table 3), transmembrane transporter activity (*Oca2*, *Slc26a8*, *Slc6a13*, *Slc17a6*, *Slc38a2*, combined score 8.42) (Supplementary Table 4), and for lipase activity (*Cckbr*, *Pla2g4e*, *Enpp2*, *Pnpla3*, combined score 6.2) (Supplementary Table 4). Notably, a recent study also reported upregulation of other lipid metabolism related genes in miRNA depleted cones^16^ (*Dhcr24*, *Insig1*, *Npc2*). Gene set enrichment analysis (GSEA) was also performed to analyse changes of gene sets rather than individual genes. Using the differentially expressed genes ranked by fold change one negatively correlated and seven positively correlated gene sets were identified in the Dicer CKO (see Supplementary Table 5). The latter upregulated gene sets included apoptosis, which may be relevant for the cone loss, as well as TGF-β-signalling and Wnt-β-catenin signalling.

### Upregulated predicted targets and miRNA sequencing in Dicer CKO

Finally, to investigate the molecular networks directly affected by the lack of Dicer we sought to identify changes in specific miRNAs in the Dicer CKO retina by RNAseq analysis at P21 and 3.5 months of age. We found that the seven miRNAs previously described as being most highly expressed in cones^16^ (miR-182-5p, miR-181a-5p, miR-183-5p, miR-181b-5p, miR-let-7f, miR-26a-5p and miR-191-5p) were amongst the most highly expressed in the whole retina for both control and mutant P21 and 3.5 months old retinas. However, these did not show significantly altered expression in the Dicer CKO retina. qPCR analysis for miR-182; miR-181a; miR-183, reported as the top three cone miRNAs^16^ indicated a non-significant trend of downregulation (0.75 cf. control; n = 3, p > 0.05) for all three miRNAs when compared to wild type controls.

Unsupervised clustering analysis of the miRNAseq data showed P21 control and Dicer CKO samples clustering together away from the 3.5 month-old control and Dicer CKO sample, which also clustered together (Fig. 7H). 103 and 72 miRNAs were significantly differentially expressed between control P21 and 3.5-month samples and between mutant P21 and 3.5 month samples, respectively. While 57 miRNAs overlapped, we found that 25 were only upregulated in controls from P21 to 3.5 months, including miR-96-5p from the sensory cluster. Conversely, 9 miRNAs were exclusively downregulated in the CKO retina from P21 to 3.5 months, including miR-182-5p from the sensory cluster. Consequently, these miRNAs could be contributing to the observed phenotype and are potential candidates for promoting cone survival. However, when expression levels were compared directly between P21 control and CKO samples no miRNAs showed significantly different levels and between 3.5 months old control and CKO samples, only miR-184-3p was significantly upregulated (fold change of 2.72).

We also performed target predictions for miR-182-5p, miR-181a-5p, miR-183-5p, miR-181b-5p, miR-let-7f, miR-26a-5p and miR-191-5p using three prediction algorithms (miRWalk, miRanda and Targetscan). Only upregulated targets predicted by all three were considered, since miRNAs are generally considered to be negative regulators of gene expression. We found that 13 of the upregulated genes (Table 1) were predicted targets of these miRNAs. Considering only the 11 genes that were significantly upregulated in Dicer CKO retinas and that were expressed in cones (from our cone RNAseq data) (Fig. 7E), *Cckbr*, which encodes a cholecystokinin receptor group G-protein coupled receptor was found to be a predicted target of miR-183 (Table 1) and has been previously reported to be expressed in cones^32,42^.

**Table 1:** miRNA target prediction

miRNAs	Predicted targets upregulated in Dicer KCO
miR-182-5p	Fam110a, Wisp1, Kif19a, Eif3j2
miR-183-5p	**Cckbr**
miR-181a-5p	Cd8a, Tm7sf3, Fzd10, Wisp1, Eif3j2
miR-181b-5p	Cd8a, Tm7sf3, Fzd10, Wisp1, Eif3j2
Let-7f-5p	Wnt9a
miR-26a-5p	Inhbb, Chst3
miR-191-5p	none
TOTAL	11 unique targets including one also expressed in cones

miRNA target prediction. Predicted targets for the seven most highly

expressed miRNAs in cone photoreceptors that are upregulated in Dicer CKO P21

whole retinas. Only Cckbr is also expressed in cones. Predicted targets of selected miRNAs that are upregulated in Dicer CKO retinas.

## Discussion

In the present study we present data indicating that *Dicer1* is needed for the survival of postmitotic cone photoreceptors. We were not able to detect Dicer protein with cell specific resolution in wild type retina in order to evaluate whether the conditional Cre mediated disruption of the *Dicer1* locus removes Dicer protein from cones. However, as we were able to show clear deletion of the viable *Dicer1* allele in retina as detected by genomic and RT PCR in the Dicer CKO retina and not in control retina we reasoned that the observed phenotypes are due to loss of *Dicer1* function. *Dicer1* conditional knockout retinas appear to develop properly but undergo early onset cone degeneration. This cone degeneration is characterised by initial segment impairment and progressive cone loss, and is accompanied by defective cone function. However, loss of cone photoreceptors did not affect rod survival or function, and therefore the degeneration described here is reminiscent of a cone dystrophy. These findings suggest that miRNAs are involved in the survival of cone photoreceptors. Of interest is the idea that miRNA dysregulation may also contribute to the pathologies described in other inherited cone dystrophies.

The Dicer CKO mice also displayed some loss of inner retinal neurons, in particular ganglion and horizontal cells. Our observation of early Chrnb4-cre activity in embryonic RPCs is in line with a recent study in which Chrnb4-GFP expression was reported in new born cones and early RPCs^33^, and suggests that lack of Dicer may affect descendants of these progenitors, namely early born cell types such as ganglion and horizontal cells. The fact that retinal laminar organisation was generally unaffected in the Dicer CKO, apart from the severe effect on cones, suggests that the observed phenotype was due to the cell autonomous effect of loss of Dicer in specific cone cells. Nevertheless, the Chrnb4-cre driver activity was not sufficient to lead to loss of all cones within the time period investigated (up to 6 months). In future studies to confirm that the cone effects are due to cell autonomous loss of Dicer, either a cone-specific Cre allele is needed, or rescue of the phenotype with a cone-specific construct that expresses Dicer is required.

The role of miRNAs in rod survival has already been demonstrated in some studies, whether it was caused by the constitutive loss of the miR-183 cluster^18,19^ or by the global loss of miRNAs in postmitotic rods^29^. On the other hand, how miRNAs are involved in cone survival remains unclear. Knockout of miR-124a during retinal development led to some premature cone cell death at early postnatal stages and loss of M-opsin in adult mice^23^. Knockout of miR-21, one of the most abundant miRNAs in the eye, caused a reduction in cone density to 50% of controls by 18 months^45^. However, global miRNA loss in cones via DGCR8 knockout led to outer segment impairment, but not to survival defects^16^. In this latter study, complete recombination only occurred after 2 months and retinas older than 3 month old were not analysed^16^. In our study, we observe Cre recombination activity in some newly born cone precursors and it is therefore possible that miRNAs are needed for the maturation and survival of immature cones. In this study we could not distinguish between the delayed effect of early loss of Dicer from delayed recombination, or whether there was disrupted maturation of some segments. Single cell analysis of cone maturation markers and recombination could help resolve this question. Two recent studies^46,47^ showed that depletion of miR-183/96^47^, or the miR-183 cluster^46^, in mice resulted in cone and rod postnatal maturation abnormalities and ERG defects, accompanied by an early progressive rod degeneration and followed by later cone survival defects. Our study showed early onset cone cell death, independent of rod survival, and suggests that additional miRNAs to the cluster family may be involved in maintaining cone homeostasis and promoting their survival.

Loss of DICER1 and DGCR8 in RPE cells led to survival and functional defects of the RPE^48^ and subsequent photoreceptor outer segment impairment^49^. In our study Cre activity was not detected in RPE cells, suggesting that the observed cone loss and outer segment defects was not due to RPE defects. RPE defects have also been attributed to the accumulation of toxic Alu RNA^50,51^, although accumulation of these Alu elements was not reported in rods after Dicer knockout^29^, or in some Dicer RPE knockout models^48,49^ and we did not detect increased Alu RNA in RNAseq analyses.

ERG analysis revealed impaired photopic function, consistent with the loss of cones. While rod scotopic a-wave amplitudes did not seem to be affected, reduced scotopic b-waves were observed. This could be due partially to the loss of cones and could also reflect abnormalities in inner cells. The peak frequency of the rod OPs in this study was 107 Hz, which agrees with the range 100–120 Hz described in wild-type C57BL/6*J* mouse by Lei *et al*.^52^. The amplitudes and peak times of the scotopic OPs were broadly similar when overlaid (Fig. 6I) which suggests that the Dicer CKO does not impair rod signaling pathways to the spiking neurons of the inner retina. The photopic OPs peak frequency occurred at 57 Hz in both control *Dicer*^*flox/flox*^ or *Dicer*^*flox*/+^ and Dicer CKO mice. This is similar to that reported by Yu and Peachey^53^ and earlier than 70–85 Hz reported in wild-type C57BL/6*J* mouse^52^. The cone OP amplitudes were 40–50% smaller in the Dicer CKO mice, but peak times were similar (Fig. 6H). This likely reflects a proportional loss of cone driven signals. The unchanged OP timing in the Dicer CKO mice suggests the cone signalling pathways in the inner retina are relatively unimpaired.

Transcriptome analysis of whole retinas at P21 revealed some changes between control and Dicer CKO mice in line with the modest changes reported in a rod-specific Dicer knockout mice^29^. As cones only account for 3% of the whole retina^36^ some changes in gene expression occurring between control and mutant could be masked and/or under represented if the genes in question were also expressed in other more abundant cell types such as rods, amacrine or bipolar cells. Technically it proved not possible to consistently isolate sufficient quantities of high quality RNA from isolated Dicer CKO cones sufficient to perform mRNA or miRNA sequence analysis, due to small litter sizes, and low cell and RNA yields after sorting. Nevertheless, in total neural retina analysis we observed downregulation of cone genes such as *Opn1sw*, *Kcne2* and *Gulo*, as well as other genes expressed in cones. In DGCR8 knockout mice, a loss of a cone signature was previously reported prior to outer segment loss^16^. Surprisingly significant changes were observed in groups of genes associated with expression in the RPE or the lens, rather than cone cells. These included, four downregulated genes involved in the retinoid cycle (*Rgr*, *Rpe65*, *Lrat* and *Ttr*). In *Rpe65*^*−/−*^ and *Lrat*^*−/−*^ mice, the retinoid cycle is arrested, leading to the specific loss of cone photoreceptors, where cone opsins and cone phototransduction proteins are unable to translocate properly into the outer segments^54^. Seven crystallin transcripts were also amongst the set of downregulated genes (with *Crybb2* and *Crybb3* mRNA detected in cones); crystallins have been shown to be upregulated in many retinal degeneration models^55^ be exacerbated by the lack of α-crystallins and attenuated by their overexpression^56^, suggesting they may have a protective role during retinal degeneration against oxidative stress.

We found that 11 of the 39 significantly upregulated genes in Dicer CKO retina were expressed in cone photoreceptors and are therefore candidates of being directly regulated by cone-expressed miRNAs. Of these, *Cckbr* is a predicted target of miR-183 (fold change of 1.71). Another upregulated gene of interest expressed in cones, is *Agr2* (fold change of 1.96) (Anterior gradient 2), which has been previously shown to be activated by NeuroD1^57^. This gene encodes for a protein disulfide isomerase (PDI), which has been shown to be critical for protein folding in the endoplasmic reticulum (ER)^58^. Proteins of this family have been shown to be upregulated in neurodegenerative processes as an adaptive response to ER induced stress and thus conferring protection against neuronal cell death^59,60^.

In an attempt to elucidate those cone-expressed miRNAs that could be contributing to the observed cone dystrophy phenotype, miRNA sequencing analysis was performed on neural retina from P21 and 3.5 month old mice. At the read depth used no miRNAs were found significantly downregulated in Dicer CKO retinas at either timepoint. However, miR-184 was significantly upregulated in Dicer CKO. Overexpression of miR-184 in neuroblastoma lines has been linked with increased apoptosis^61^. In addition its downregulated expression has been reported in the RPE of AMD patients^62^. Inhibition of miR-184 in ARPE-19 cells led to a decrease in the ability of these cells to uptake photoreceptor outer segments via phagocytosis partially due to increased levels of its target ezrin^62^. Downregulation of miR-184 and subsequent upregulation of its target frizzled-7 from the Wnt pathway was also reported in mouse models of oxygen induced retinopathy^63^. The lack of significantly downregulated cone-enriched miRNAs, such as the sensory cluster, in Dicer CKO retinas, may be due to their pan-retinal expression masking changes specifically in cone cells; as these miRNAs are also expressed in the more abundant rod cells^16,17^. Or the phenotype may be due to the effect of depletion of multiple low abundance miRNAs. By extension, these data suggest that there are no very highly expressed cone-specific miRNAs whose depletion causes the observed phenotype. These data also indicate that the observed cone dystrophy following conditional Dicer ablation did not lead to a major dysregulation of the pan-retinal miRnome.

These findings demonstrate that *Dicer1* activity is essential for the survival of cone photoreceptors and suggest that miRNAs may play an important role in the pathology of cone photoreceptor dystrophies via a number of pathways. Further profiling of miRNAs in models of photoreceptor degeneration may provide important markers of retinal disease. Elucidating which miRNA-mRNA networks promote cone survival will be important for developing miRNA-based therapies to ameliorate sight loss.

## Materials and Methods

### Animals

*Chrnb4-cre; Dicer*^*flox/flox*^ conditional knockout mice (CKO) were generated by crossing *Chrnb4-cre*, C57BL/6 transgenic mice (generated by Nathaniel Heinz as part of the Gensat project^31^; Stock # 0362303-UCD; Tg(Chrnb4-cre)OL57Gsat/Mmucd), with *Dicer*^*flox*^, C57BL/6, mice (obtained as a kind gift from Prof Matthias Merkenschlager^64^ and Prof John Wood; with Dicer exons 20–21 flanked by LoxP sites). Specifically, *Chrnb4-cre* mice were crossed with *Dicer*^*flox/+*^ mice in order to obtain *Chrnb4-cre; Dicer*^*flox*/+^. The mice used for analysis were obtained by crossing *Chrnb4-cre; Dicer*^*flox*/+^ females with *Dicer*^*flox/flox*^ males, obtaining control *Dicer*^*flox/flox*^ or *Dicer*^*flox*/+^ control mice as well as Dicer CKO *Chrnb4-cre; Dicer*^*flox/flox*^ mice.

*Chrnb4-cre; R26*^*YFP*/+^ were obtained by crossing *Chrnb4-cre* female mice with *R26*^*YFP*/+^ C57BL/6 males^34^. All mice were housed at University College London animal facilities and all experiments were conducted in agreement with the Animals (Scientific Procedures) Act 1986 after UCL Ethics Review and UK Home Office licencing and all experiments were performed in accordance with these regulations.

For each timepoint and genotype, at least n > 3 mice were used for analysis. *Dicer*^*flox/+*^/*Dicer*^*flox/flox*^ littermates of Dicer CKO mice (*Chrnb4-cre*; *Dicer*^*flox/flox*^ mice) were used as controls throughout (Figures 2–7). Genotyping PCRs were performed from ear biopsies, using Taq DNA Polymerase from Qiagen (201203) according to manufacturer’s instructions. PCR to show recombination of *Dicer1* locus was performed on DNA extracted from neural retina and tail of *Dicer*^*flox/flox*^ and *Chrnb4-cre; Dicer*^*flox/flox*^ mice; the 309 bp KO band was only detected in neural retina of the latter. The following primers were used for genotyping:

**Table:** 

Genotyping Primers	Primer sequence (5′-3′)	Amplicon size
GFP	Fw: CTACGGCGTGCAGTGCTTCA Rv: TTCTGCTGGTAGTGGTCGGC	GFP band: 500bp
Dicer (*x & z*)	31831: AGTGTAGCCTTAGCCATTTGC 32050AS: CTGGTGGCTTGAGGACAAGAC	Wt band: 259bp Lox band: 390bp
Dicer KO (*y & z*)	28290: AGTAATGTGAGCAATAGTCCCAG 32050AS: CTGGTGGCTTGAGGACAAGAC	KO band: 309bp
R26^YFP^	R1: AAAGTCGCTCTGAGTTGTTAT R2: GCGAAGAGTTTGTCCTCAACC R3: GGAGCGGGAGAAATGGATATG	Wt band: 500bp YFP band: 250bp
Cre	CreA: GATGCAACGAGTGATGAGGTTCGC CreB: ACCCTGATCCTGGCAATTTCGGC	Cre band: 500bp

### Immunohistochemistry

Enucleated mouse eyes were fixed in 4% paraformaldehyde. Embryonic tissue was fixed for 45 minutes at room temperature. Postnatal eyes were fixed for one hour at room temperature. Washes were performed in Phosphate-buffered saline (PBS), three times for 5 minutes at 4 °C. The anterior segment of postnatal eyes was then removed. Eyes were then incubated in 30% sucrose for 2 h at room temperature for cryo-protection, embedded in optimal cutting temperature (OCT) compound (RA Lamb), frozen in dry ice and stored at −80 °C. Serial tissue sections were prepared using a cryostat (Leica CM1900 UV), with a thickness of 10–14 μm and collected on Superfrost^TM^ plus glass slides (VWR). Slides were kept at −80 °C.

For immunohistochemistry, tissue slides were air dried for 15 minutes at room temperature, OCT compound was removed in PBS for 15 minutes at 37 °C. Tissue sections were blocked in blocking solution consisting of 1% bovine serum albumin (BSA) in PBS pH7.4 containing 0.1% Triton X-100; for one hour at room temperature before proceeding to primary antibody incubation. Primary antibodies were diluted in blocking solution to the desired concentration. The following primary antibodies were used: Cone arrestin (Millipore AB15282, 1:5000) as a marker of cones^65^, RxRg (Abcam AB15518, 1:200) as a marker of cones^35^, BRN3A (Millipore MAB1585, 1:300) as a marker of ganglion cells^66^, AP-2 (DSHB 3B5-S, 1:200) as a marker of amacrine and horizontal cells^67^, RHO (Sigma 04886, 1:1000) for rods^68^, CHX10 (Chemicon AB9016, 1:200) as a marker of RPCs^69^ and bipolar cells^70^, ONECUT1 (Santa Cruz sc-13050, 1:400) for horizontal cells^71^, PAX6 (Covane PRB278P, 1:300) for inner retinal cells^72^, OTX2 (Abcam AB21990, 1:100) for photoreceptor progenitors^26^, LM OPSIN (Chemicon AB5405, 1:500) for adult cones^65^, Cleave caspase 3 (Cell signalling technologies 9661S, 1:300) for apoptotic cells^73^ and Glial Fibrillary Acidic Protein (GFAP) (Millipore AB5804, 1:500). Primary antibodies were incubated overnight at 4 °C. Slides with no primary antibody were used as negative control. After incubation, tissue slides were washed in PBS at 4 °C, three times for 5 minutes. The corresponding secondary antibody, diluted 1:800 in blocking solution, was then added for 1 hour at room temperature (Goat anti-rabbit AlexaFluor 488, Invitrogen A31556 and 594, Invitrogen A11037; and the Goat anti-mouse AlexaFluor 488, Invitrogen A1080, and 594, Invitrogen A21044). For GFP/YFP staining, conjugated goat anti-GFP (FITC) antibody was used (Abcam AB6662, 1:200) at this stage. Tissue slides were then washed in PBS at 4 °C, three times for 5 minutes, incubated for 5 minutes at room temperature in DAPI (1:3000, Sigma-Aldrich) for nuclear staining and washed in PBS at 4 °C, three times for 5 minutes prior to cover-slipping with Hydromount medium (National Diagnostics HS-106).

For toluidine blue staining eyes were fixed overnight in Karnovsky’s medium (2% PFA and 2.5% Glutaraldehyde in 0.1 M sodium cacodylate buffer). After fixation samples were washed in PB (phosphate buffer) and fixed again for one hour in 1% osmium tetroxide in 0.1 M phosphate buffer. A series of dehydration steps was then carried out: 50% ethanol for 15 minutes, 70% Ethanol for 15 minutes, 95% Ethanol for 15 minutes, 100% Ethanol 2 × 15 minutes, 100% Propylene oxide for 5 minutes, 100% Propylene oxide for 5 minutes and 100% Propylene oxide for 10 minutes. Samples were then incubated overnight in resin and propylene oxide in a 1:1 ratio and then in resin for seven hours. Samples were then embedded in beam capsules and baked overnight at 60 °C. Sections were then cut semithins with 700 nm thickness and stained with toluidine blue for two minutes and washed in distilled water.

### Microscopy

Confocal images were acquired as using a Zeiss LSM710 (Zen2009, Zeiss) and processed using FIJI, Photoshop CS6 (Adobe) and Illustrator CS6 (Adobe). Double labelling analysis was performed on FIJI.

### Cell counting and measurements

Cell counting was performed on images acquired using confocal microscopy using a X20 objective. For each genotype and each cell type (labelled with specific antibody), three different sections were analysed (sagittal section with the optic nerve visible, and parasagittal sections before and after the optic nerve) per eye. From each tissue section, 2 or 3 images of peripheral retina and 2 images of central retina were acquired for counting using FIJI. The number of cells counted per mm of retinal length was averaged for all 3 sections for each mouse, distinguishing peripheral and central retinas. At E17, the number of positive cells was normalised per mm length of the neuroblastic layer (NBL) (measured with FIJI). Cleaved caspase-3 cells were normalised to the whole counted section (due to its very low presence). For P21, the number of counted cells was normalised relative to the length of either the outer nuclear layer (ONL) (for cone arrestin cells), or the inner nuclear layer (INL) (for Onecut1, AP-2 and Chx10 cells), or the ganglion cell layer (GCL) (for Brn3A cells). Measurements of the thickness of the NBL, ONL and INL were taken on DAPI stained images. For each mouse, 3 tissue sections were analysed. From each tissue section 2–3 peripheral images and 2 central retina images were analysed. For each image, 6 measurements were averaged for the NBL, ONL and INL (36–54 measurements per eye for the periphery and 36 for the central respectively per eye). Measurements of the segment length of cone cells were acquired from CA stained sagittal sections. From one tissue section, 60–200 cells were analysed per animal. For each field of view, the segments (inner and outer segments) of all visible cones in the captured image were measured from the basal side of the inner segment (IS) to the apical side of the outer segment (OS). If no segments were detected for a given CA^+ve^ cell body, the segment length was recorded as 0 μm.

Statistical analysis on cell counts was performed using a non-parametric unpaired Mann Whitney test with Prism (GraphPad software). Data is represented as mean ± SEM, comparing control and Dicer CKO.

### qRT-PCR

Total RNA was extracted from neural retinas using the miRNeasy Micro Kit from Qiagen (217084) following manufacturer’s instructions. The optional DNase treatment was also included to remove genomic DNA traces from each sample. The eluted total RNA was retrotranscribed into cDNA using the miScript II RT Kit (Qiagen 218160). Expression of mmu-miR-183-5p; mmu-miR-182-5p and mmu-miR-181a-5p was assessed using miScript Primer assays (Qiagen) together with the miScript SYBR Green Kit (Qiagen 218073) on a 7500 Real-Time PCR System according to manufacturer’s recommendations. Relative miRNA expression data was normalised using RNU6 as an endogenous reference. The Livak 2^∆∆Ct^ method was used^74^, normalising the expression levels to control wild type samples. Statistical analysis on the relative fold expression was performed using a non-parametric unpaired Mann Whitney test with Prism (GraphPad software). Data is represented as mean ± SEM, comparing control and Dicer CKO. Expression of Dicer mRNA was assessed using cDNA prepared using the miScript II RT Kit (Qiagen 218160) and PCR performed using the MyFi DNA Polymerase kit (Bioline Bio-21117) and primers: 5′ GAGAAGCCTGCCCTGGAGTTTA (exon 16), 5′ TCAACGGCTTTGCTAGGATCCA (exon 23) for *Dicer* and 5′ ATG ACA TCA AGA AGG TGG T (exon 6), 5′ CAT ACC AGG AAA TGA GCT T (exon 6) for *Gapdh*. PCR products were run on a 1% agarose gel and visualised by SYBR Safe. Expected amplicons are 1007 bp for *Dicer* ∆20–21 and 177 bp for *Gapdh*.

### RNA-seq

For each control (n = 3) and each Dicer CKO (n = 3), the neural retinas of the right eye were dissected excluding RPE, optic nerve, lens and anterior segment tissue. Total RNA was then extracted using the miRNeasy Micro Kit from Qiagen (217084) following manufacturer’s instructions and including DNase treatment. RNA quality and concentration was measured using the TapeStation (Agilent). RNA library preparations were generated using the Illumina TruSeq Stranded mRNA Library Prep Kit (RS-122-2103). Sequencing was pair-ended, 75 bp, with 15 million read depth and an Illumina TruSeq4000 was used. The sequenced files were processed for data analysis using the XploreRNA tool from Exiqon, whose NGS pipeline is based in the Tuxedo software package and includes the following component: Bowtie2 (v2.2.2), Tophat (v2.0.11) and Cufflinks (v2.2.1). Transcript assembly was performed using Cufflinks and Cuffquant. Normalization and supervised differential expression analysis was performed using Cuffnorm and Cuffdiff. Normalised gene expression levels are shown as Fragments per Kilobase of transcript per Million mapped reads (FPKM). FPKM were averaged for all three controls and all three mutants. q-value below 0.05 was considered as significant. q-values represent p-values adjusted using the Benjamini-Hochberg False Discovery Rate (FDR) approach to correct for multiple testing. Enrichr^75,76^ was used for gene ontology (GO) enrichment of significantly upregulated and downregulated genes. For Gene Set Enrichment Analysis (GSEA), genes were ranked according to log2 Fold Change (Mutant vs WT), genes were converted to their human orthologues and pre-ranked GSEA was run using the 50 Hallmark curated gene sets from the Molecular Signature Database^77^ and a False Discovery rate threshold of FDR < 0.25 as recommended^78^.

Chrnb4-GFP neural retinas were dissected in EBSS and dissociated using the Papain Dissociation System (Worthington Biochemical) following manufacturer’s instructions and as previously described^79^. Dissociations were carried for *Chrnb4-GFP* retinas at postnatal stages P8 and P42 and up to eight retinas were pooled per dissociation. Following retinal dissociation and before FACS sorting, rods were stained for CD73-PE (eBioscience clone TY/11.8) as previously described^79^. After incubation, cells were resuspended in cold PBS, 1% FBS prior to FACS sorting. Cell sorting was carried out for the isolation of Chrnb4-GFP^+ve^ cones and CD73-PE^+ve^ rods using a BD FACS Aria III. Gating for CD73-PE and Chrnb4-GFP were determined for each experiment using the corresponding controls. For the P8 stage, only the Chrnb4-GFP^High^ population was isolated. The SMARTer-seq low input RNA kit from Clonetech (High Vol-cat 634828) was used for library prepping and input sample was normalised to the limiting sample. An Illumina HiSeq4000 sequencer was used and the parameters were 75 bp, pair ended and 15 million read depth. Analysis was also performed using the XploreRNA tool (Exiqon) as described above. Venn diagrams were performed using the Bioinformatics & Evolutionary Genomics online tool (http://bioinformatics.psb.ugent.be/webtools/Venn/). Genes with normalised FPKM values higher than 5 (FPKM > 5) in P8 and P42 cone samples were selected to define a cone transcriptome of 7797 unique genes. Accession number for the RNAseq data reported in this paper is ArrayExpress: E-MTAB-6133.

### Small RNAseq

Small RNA sequencing was performed on whole neural retinas. For all 12 samples, the neural retinas of the right eye were dissected. Total RNA was then extracted using the miRNeasy Micro Kit from Qiagen (217084) following manufacturer’s instructions and including DNase treatment. Library preparation was carried out using the NEBNext Multiplex Small RNA Library Prep Set for Illumina. An Illumina Miseq sequencer was used, with single reads. FASTQ files were then uploaded to the XploreRNA tool from Exiqon for automated analysis. Sequenced reads were aligned to the sequences of mature miRNAs and to the reference genome using Bowtie 2 (version 2.2.6). The differential expression analysis was performed using EdgeR. Unsupervised clustering and all visualisations were performed by Exiqon. Accession number for the small RNAseq data reported in this paper is ArrayExpress: E-MTAB-6132.

### Electroretinogram

ERGs of 4 months old mice were recorded using an Epsion E^2^ system with a ColourDome simulator (Diagnosis LLC, Lowell, MA) as described in Pearson *et al*.^45^. Both eyes of four control mice (*Dicer*^*flox/flox*^ or *Dicer*^*flox*/+^) (n = 4) and three Dicer CKO mice (n = 3) were tested. A masked protocol was employed so that the person performing the ERGs and the analysis did not know which mice were control and Dicer CKO. Scotopic and photopic ERGs were carried for each animal. Animals were dark-adapted overnight and the scotopic ERG tests were performed first for every animal under dim red light followed by photopic tests. Animals were anaesthetised and kept warm with a thermostatically controlled heat wrap. Pupils were dilated using Tropicamide 1%. Corneal contact electrodes as well as midline subdermal reference and ground electrodes were placed. Viscotears were then placed on each cornea to keep them moist during testing. ERG responses were obtained simultaneously from both eyes. For scotopic assessments, continuous flash recordings were obtained at light intensities of 0.000001, 0.00001 and 0.0001 cds/m^2^ and single flash recordings were obtained at light intensities of 0.001, 0.01, 0.1, 1, 10, 31.6, 75.28 cds/m^2^ using a sampling frequency of 5 kHz, a pulse period of 4 ms, and a decreasing frequency stimulus of 2 to 0.04 Hz. Data were recorded from 10 ms before stimulus onset to 400 ms post-stimulus. The background intensity was 0 cds/m^2^. For photopic assessments, animals were light adapted (~30 cds/m^2^) and continuous flash recordings were obtained at a light intensity of 3 cds/m^2^ using a sampling frequency of 5 kHz, a pulse period of 0 ms, and a pulse frequency of 0.5, 2, 5, 10, 15 and 30 Hz. Data were recorded from 100 ms before stimulus onset to 400 ms post-stimulus. The background intensity was 10 cds/m^2^. For analysis, the a and b wave amplitudes (a wave trough to b wave peak were measured. Due to restriction of animal movements between sites, animals could only be assessed on a single occasion. The frequency spectrum of these ERGs was analysed offline using fast fourier transform Neuroscan Scan 4.5 software (Compumedics Neuroscan, Charlotte, NC 28269, US). Photopic and scotopic oscillatory potentials (OPs) were isolated by digital 12 dB bandpass filtering. Bandpass 40–300 Hz and 60–300 Hz with zero phase shift were assessed. The trough to peak amplitudes of OP1, OP2, OP3 and OP4, when evident, were measured and the time to peaks noted.

### Experimental design

#### E17 immunostainings and countings

n = 3 *Dicer*^*flox/flox*^ control mice and n = 4 *Chrnb4-cre; Dicer*^*flox/flox*^ CKO mice were used for analysis. For each animal, three central sections were analysed, representing 3mm-5mm of retina. For the measurements of the NBL, two central sections were analysed (30 measurements for each section). Using GraphPad, a non-parametric two-tailed Mann-Whitney Test was used as a statistical test. *p* value < 0.05 was considered as significant.

#### P21 immunostainings and countings

For the number of CA^+ve^ cells, n = 5 control *Dicer*^*flox/flox*^ mice and n = 5 *Chrnb4-cre; Dicer*^*flox/flox*^ CKO mice were used for analysis. For the number of other cell types and measurements of the thickness of the ONL and INL, n = 4 control *Dicer*^*flox/flox*^ mice and n = 4 *Chrnb4-cre; Dicer*^*flox/flox*^ CKO mice were used for analysis. For each animal, three central sections were analysed, representing 3mm-5mm of retina length. Measurements of the length of CA^+ve^ segments, only the cones in the central section containing the optic nerve head were analysed (60–200 cones analysed per animal). qPCR analysis for miR-183, miR-183 and miR-181a were performed on n = 3 control *Dicer*^*flox/flox*^ and n = 3 *Chrnb4-cre; Dicer*^*flox/flox*^ CKO whole retinas. Using GraphPad, a non-parametric two-tailed Mann-Whitney Test was used as a statistical test. *p* value < 0.05 was considered as significant.

#### 3.5/4 month old analysis

For the number of CA^+ve^ cells, n = 4 control *Dicer*^*flox/flox*^ or Dicer^flox/+^ mice and n = 4 *Chrnb4-cre; Dicer*^*flox/flox*^ CKO mice were used for analysis. ONL and INL measurements were performed on n = 4 control *Dicer*^*flox/flox*^ or Dicer^flox/+^ mice and n = 4 *Chrnb4-cre; Dicer*^*flox/flox*^ CKO mice. Using GraphPad, a non-parametric two-tailed Mann-Whitney Test was used as a statistical test. *p* value < 0.05 was considered as significant. ERG analysis was performed on n = 4 control *Dicer*^*flox/flox*^ or Dicer^flox/+^ mice and n = 3 *Chrnb4-cre; Dicer*^*flox/flox*^ CKO mice. A multiple t-test was performed as a statistical test to determine significance. Multiple comparisons were adjusted using the Sidak-Bonferroni correction. Adjusted *p* value < 0.05 was considered as significant.

#### 6 month old analysis

For the number of CA^+ve^ cells, n = 3 control *Dicer*^*flox/flox*^ or Dicer^flox/+^ mice and n = 4 *Chrnb4-cre; Dicer*^*flox/flox*^ CKO mice were used for analysis. Using GraphPad, a non-parametric two-tailed Mann-Whitney Test was used as a statistical test. *p* value < 0.05 was considered as significant.

### Significance Statement

The degeneration of cone and rod photoreceptor cells, which are essential for vision, leads to irreversible blindness. Although cone photoreceptors are considerably less abundant than rods, their loss has detrimental consequences for colour vision and visual acuity. microRNAs, which regulate gene expression at the post-transcriptional level, have been linked to the aetiology of many retinal diseases, but their role in cone photoreceptor cell death remains unclear. This study indicates that DICER-mediated microRNA biogenesis is essential for cone photoreceptor survival and homeostasis.

## Supplementary information

Supplementary Figures 1–11 and Tables 1–5

## Data Availability

The data generated or analysed during this study are included in this published article. The RNAseq datasets generated during and/or analysed during the current study are deposited in the ArrayExpress repository.
