# Supplementary material for: Conditional Dicer1 depletion using Chrnb4-Cre leads to cone cell death and impaired photopic vision
Source: Sci Rep. 2019 Feb 19;9:2314. doi: 10.1038/s41598-018-38294-9 (PMC6381178; doi:10.1038/s41598-018-38294-9)
Supplement: Supplementary file 1 — Supplementary Figures 1–11 and Tables 1–5 [file 41598_2018_38294_MOESM1_ESM.docx]

**Title: Conditional *Dicer1* depletion using *Chrnb4-Cre* leads to cone cell death and impaired photopic vision**

**Authors:** Eduardo Zabala Aldunate, Valentina Di Foggia, Fabiana Di Marco, Laura Abelleira Hervas, Joana Claudio Ribeiro, Daniel L Holder, Aara Patel, Tommaso B Jannini, Dorothy A Thompson, Juan Pedro Martinez-Barbera, Rachael A Pearson, Robin R Ali, Jane C Sowden

**Title: Conditional *Dicer1* depletion using *Chrnb4-Cre* leads to cone cell death and impaired photopic vision**

**Supplementary Figure 1**

1. Genomic structure of the *Dicer* gene exons 16 to 23 in *Dicer^flox/flox^* mice, with the locations of genomic and RT-PCR primers and *loxP* sites indicated (red arrows).
2. RT PCR for Dicer mRNA using primers in exons 16 and 23 in RNA isolated from neural retina at postnatal day 21 (P21), 14 weeks of age (14W) and 6 months of age (6M) in control and Dicer CKO mice. (**B1**) A 1007bp amplicon (primers Fwd & Rev) corresponding to Dicer lacking exons 20 and 21 (Dicer ∆20-21) was detected only in retinas from *Dicerfl/fl; Chrnb4-Cre* (Dicer CKO) mice at the three timepoints indicated, and not in controls. (**B2**) Specific 177bp amplicon generated using *GAPDH* primers in all samples.
3. Recombination of floxed Dicer exons. DNA PCR showing that Dicer exons 20 and 21 were successfully recombined in Dicer CKO retinae. (**C1**) The Dicer flox amplicon (390bp) spanning a lox P site (primers y & z) was detected in all samples and WT band (259bp) detected only in *Dicerfl/+; Chrnb4-Cre* samples (Het). (**C2**) Cre amplicon (500bp) was detected in Dicer CKO and not control genomic DNA isolated from ear and neural retina at P21. (**C3**) The deleted *Dicer* allele (Dicer ∆20-21) was only detected in Dicer CKO neural retinal samples, indicating Cre recombination (309bp amplicon; primers x & z). NR: neural retina. Gel images shown in C1-3 are cropped from different parts of the same gel run.


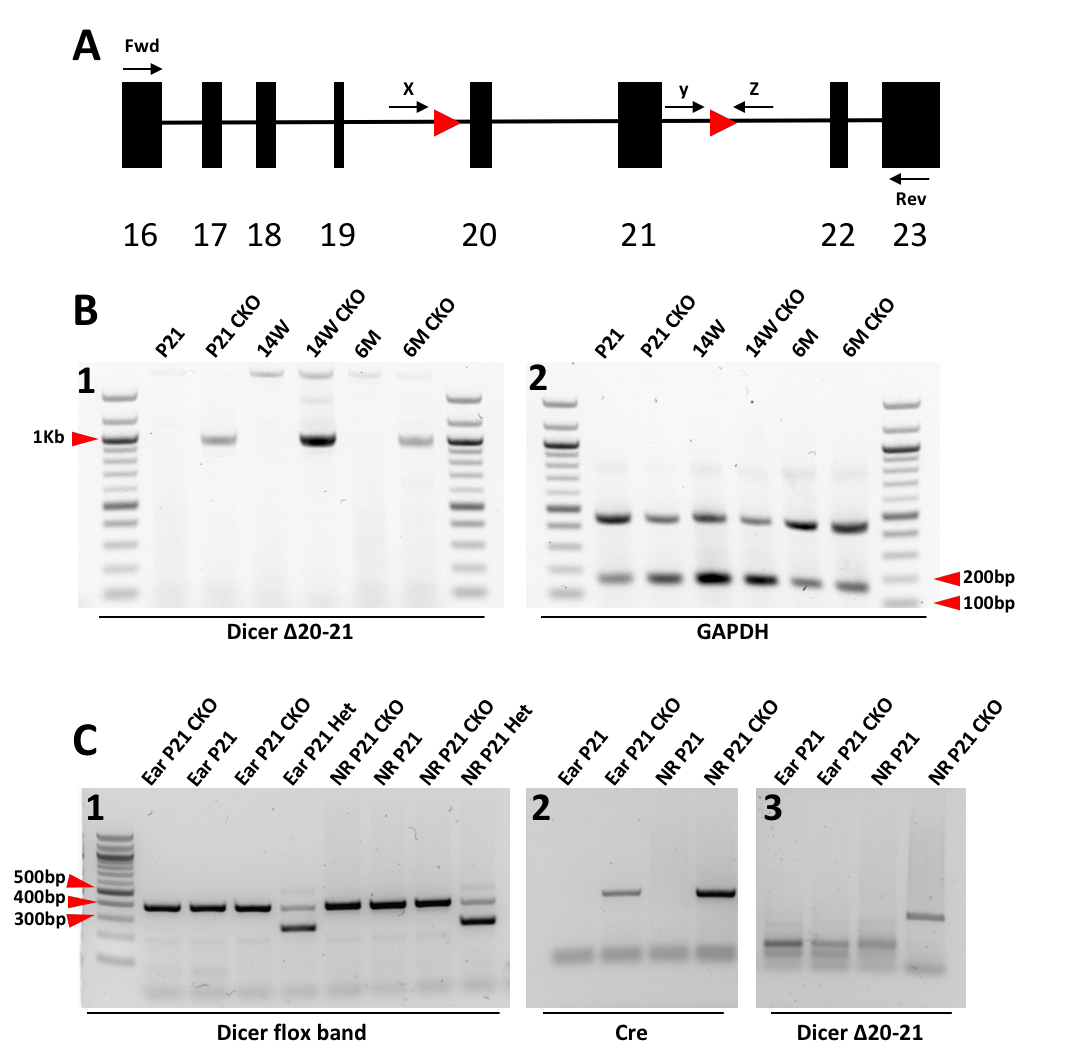


**Supplementary Figure 2**

Müller glia cell activation in CKO Dicer retina. In control, 6 month retina, GFAP (red) stained only astrocytic processes in the ganglion cell layer. The Dicer CKO retina (Chrnb4-cre; Dicer^flox/flox^ ) showed some Müller glial cell activation (8.7 ± 1.65 GFAP-labelled IPL processes per 100 μm of retinal length compared with 0.49 ± 0.49 in the control; n = 3 eyes analysed for each. Scale bars A-D: 50μm; E-F: 100μm.

**
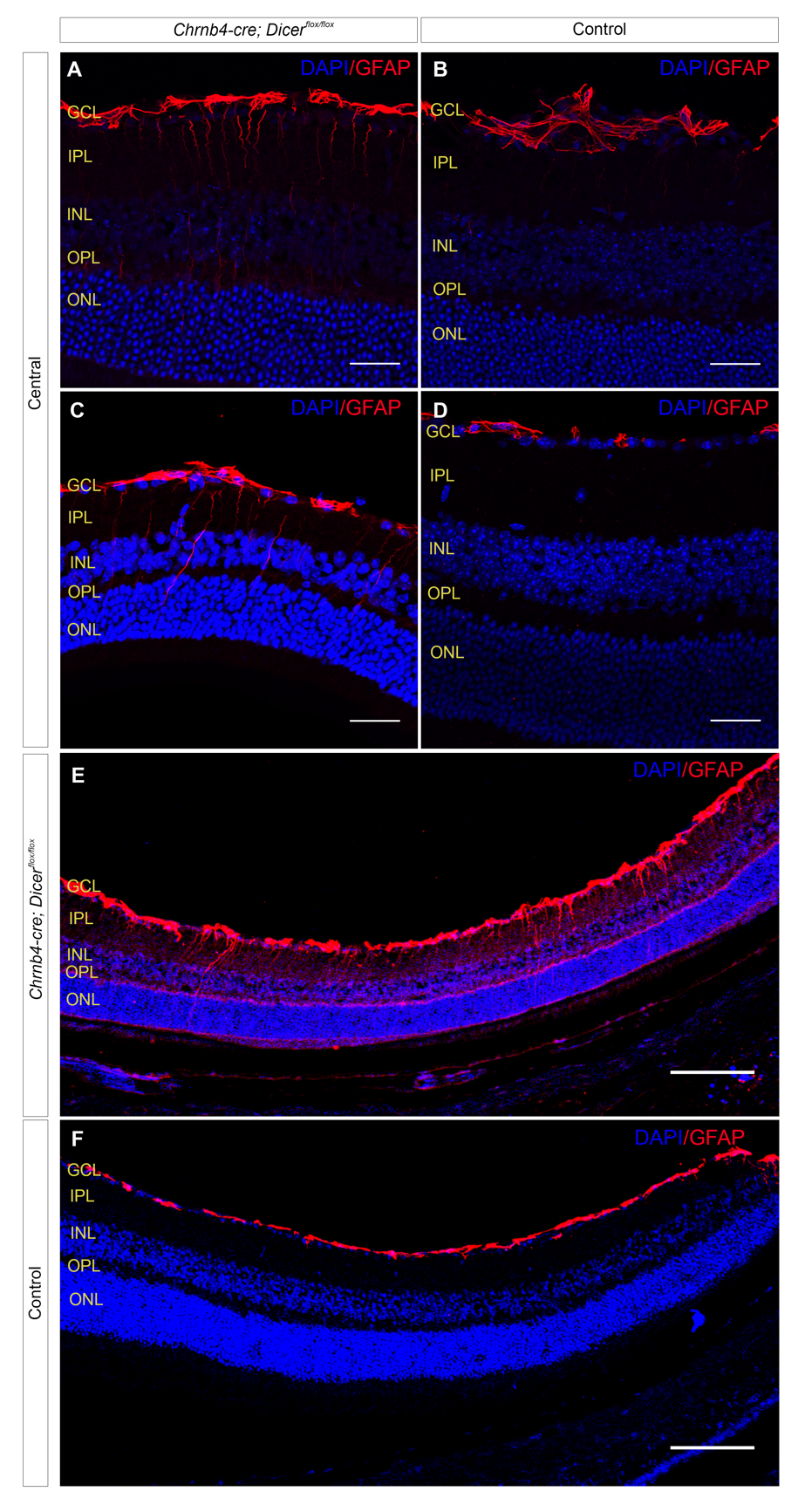
**

**Supplementary Figure 3**

Single channels shown for data presented in Figure 1A. Chrnb4-GFP, a marker of adult cone photoreceptors. Immunostaining of 6 week old *Chrnb4-GFP* retinas. Chrnb4-GFP expression (green) co-labels with the cone markers (red) CA (A) and RxRγ (B) in the outer nuclear layer (ONL). CA: cone arrestin. RxRγ: retinoid x receptor gamma. OS: outer segments. IS: inner segments. ONL: outer nuclear layer. INL: inner nuclear layer. GCL: ganglion cell layer. Scale bars: 30μm.

**
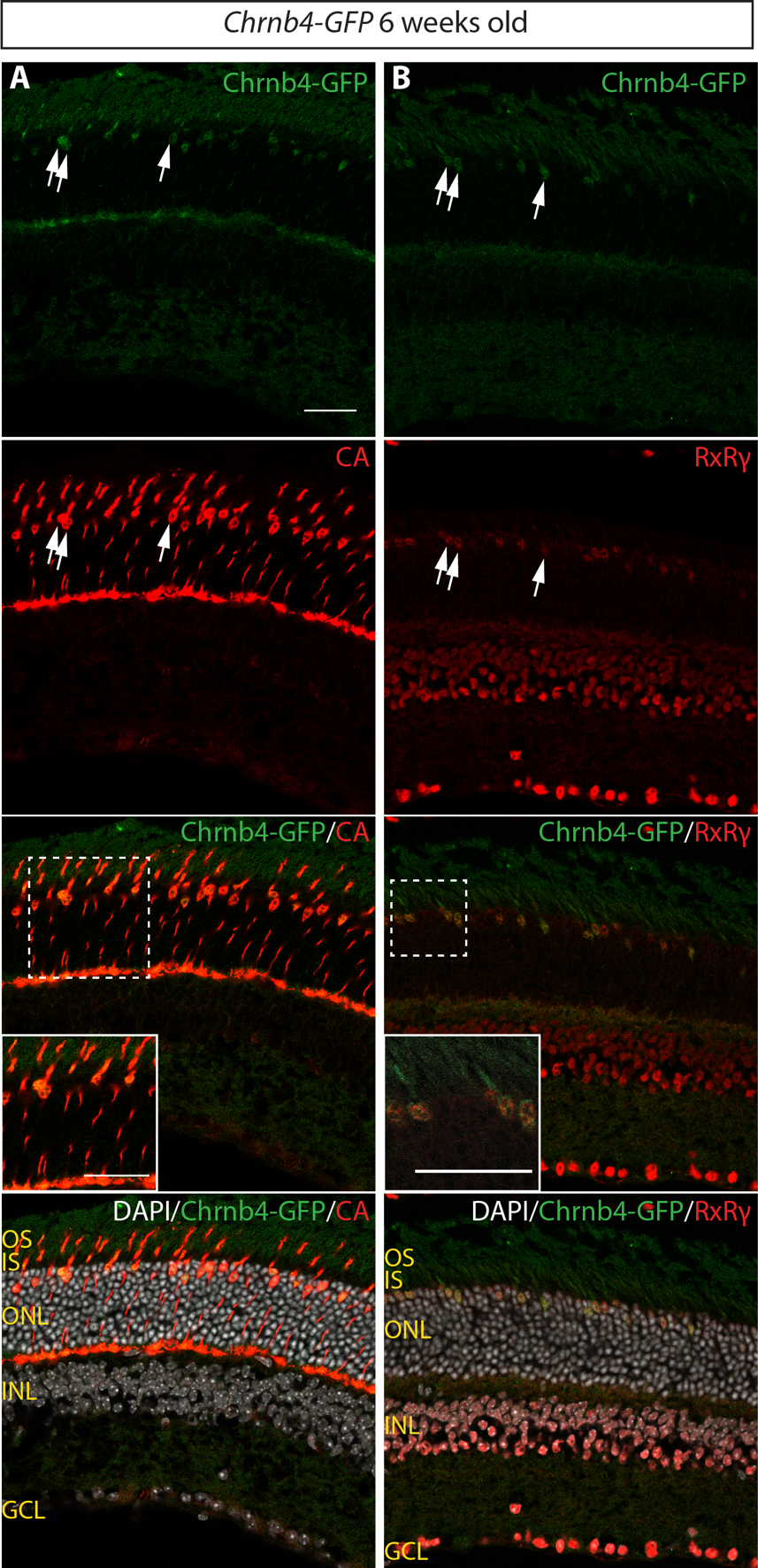
**

**Supplementary Figure 4**

Single channels shown for data presented in Figure 1B. Chrnb4-GFP, a marker of developing cone photoreceptors in the ONL. Immunostaining of postnatal day P8 *Chrnb4-GFP* retinas. Chrnb4-GFP expression (green) co-labels (white arrows) with the expression of cone markers (red) CA (A) and RxRγ (B) in the outer nuclear layer (ONL). CA: cone arrestin. RxRγ: retinoid x receptor gamma. OS: outer segments. IS: inner segments. ONL: outer nuclear layer. INL: inner nuclear layer. GCL: ganglion cell layer. Scale bar: 30μm. Scale bar of insets: 10μm. Rxrγ is a useful early cone marker based on its expression pattern in post-mitotic cones^35^; it also labels inner retinal cells.

**
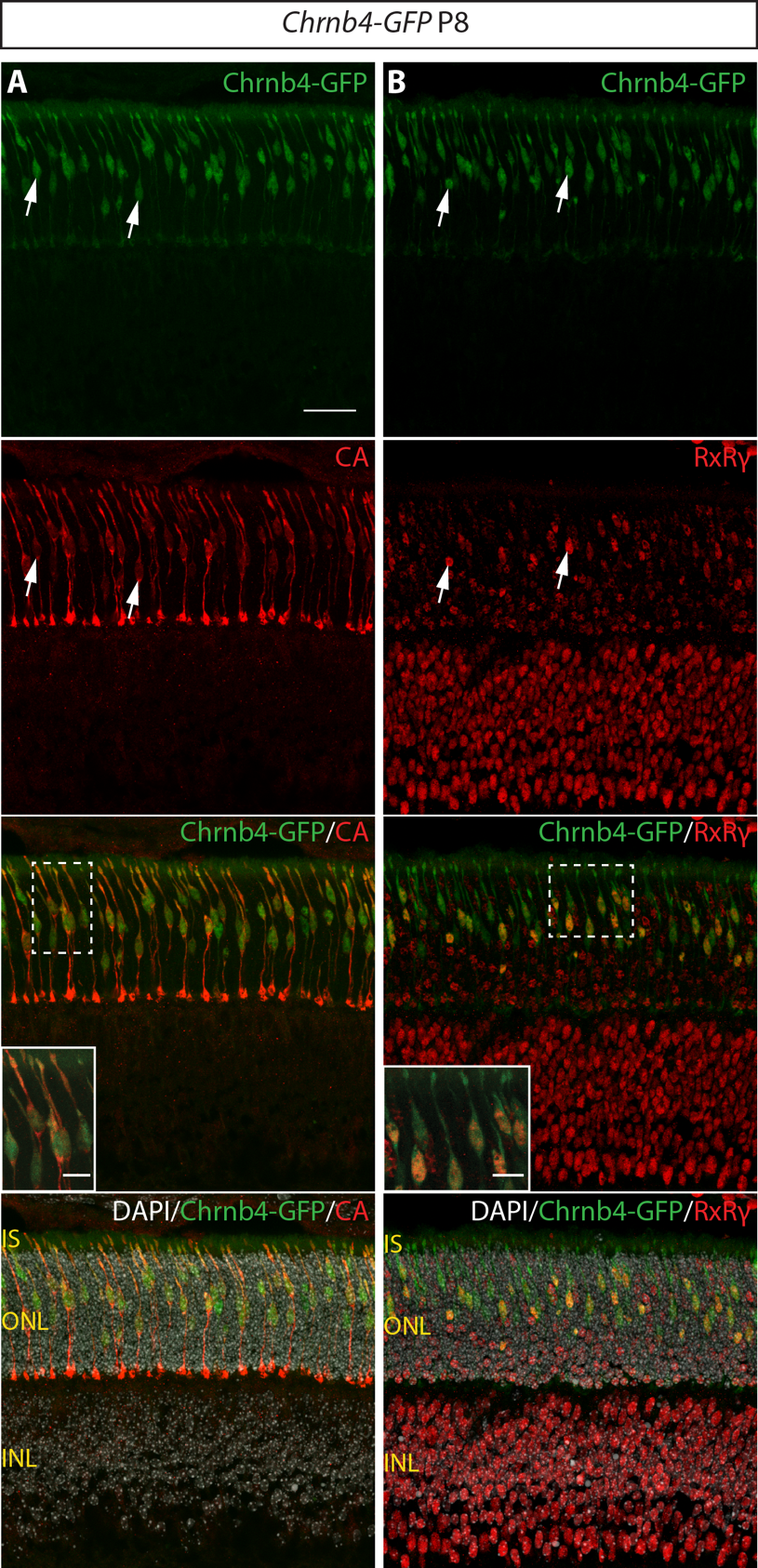
**

**Supplementary Figure 5**

Single channels shown for data presented in Figure 2D,E,G,H. Retinal histogenesis at E17. (**A-D**) Immunohistochemistry for BRN3A+ve ganglion cells in control (**A, C**) and Dicer CKO (**B, D**) developing retinas. (**E-H**) Immunohistochemistry for OTX2+ve progenitor cells in control (**E, G**) and Dicer CKO (**F, H**) developing retinas. RPE: Retinal Pigment Epithelium. NBL: neuroblastic layer. GCL: ganglion cell layer. Scale bars: 30μm. Scale bars of insets: 10μm.


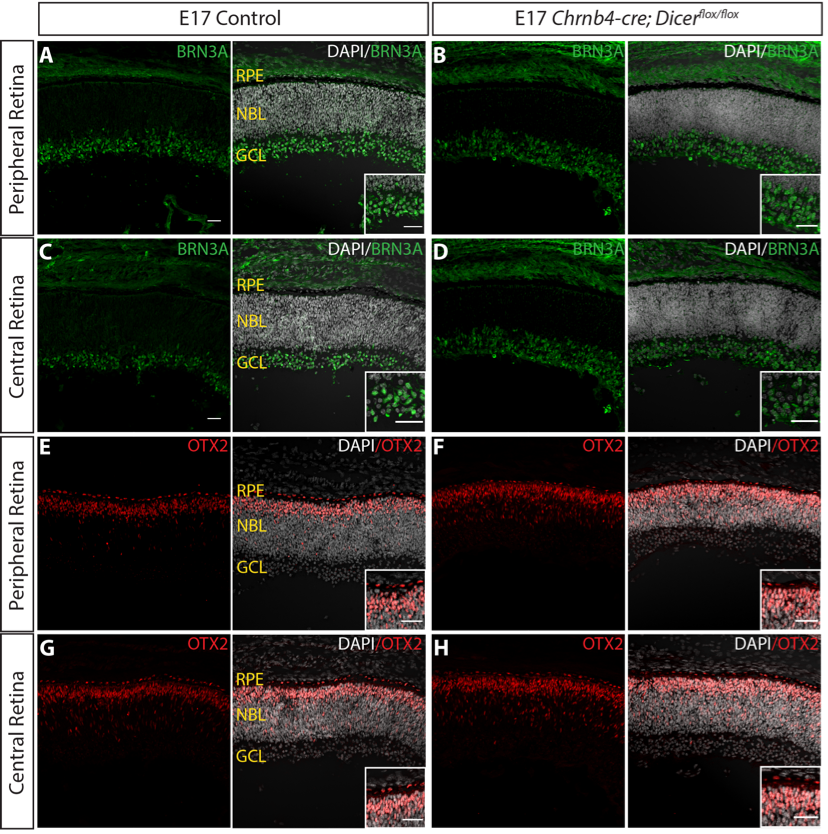


**Supplementary Figure 6**

Single channels shown for data presented in Figure 2J,K,M,N. Retinal histogenesis at E17 (continuation). (**A-D**) Immunohistochemistry for AP2+ve amacrine and horizontal cells in control (**A, C**) and Dicer CKO (**B, D**) developing retinas. (**C-D**) Immunohistochemistry for CC- 3+ve apoptotic cells in control (**E**) and Dicer CKO (**F**) developing retinas. RPE: Retinal Pigment Epithelium. NBL: neuroblastic layer. GCL: ganglion cell layer. Scale bars: 30μm. Scale bars of insets: 10μm.


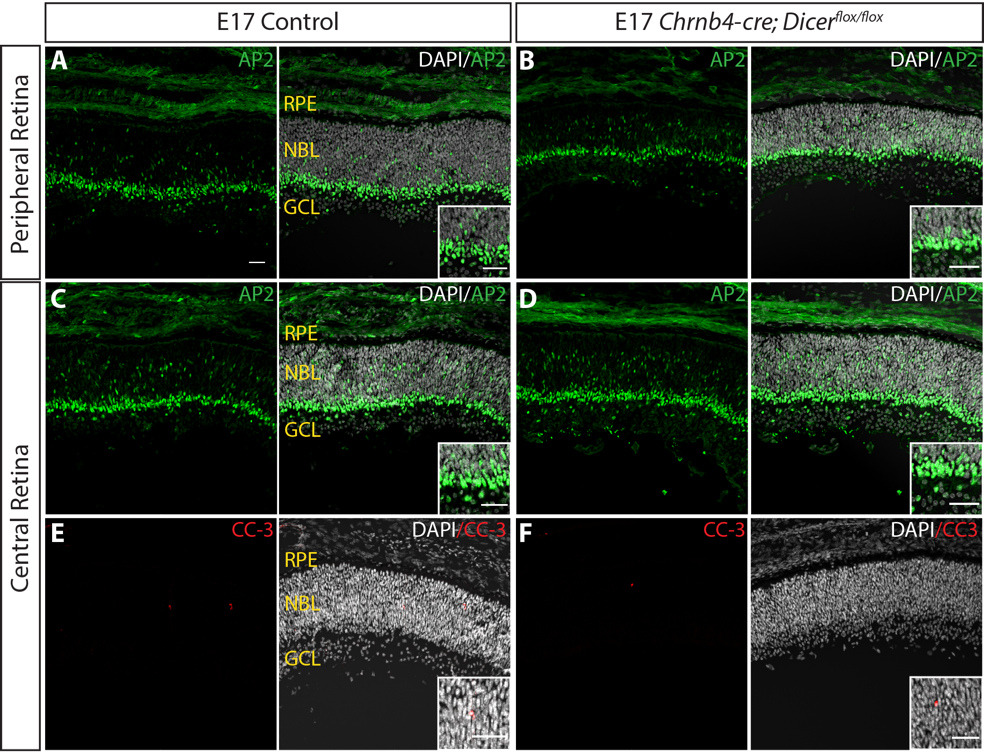


**Supplementary Figure 7**

Single channels shown for data presented in Figure 2P & Q. Cone birthing was not affected in Dicer CKO mice at E17. (**A-D**) Immunohistochemistry for early cone marker RxRγ in peripheral (**A-B**) and central (**C-D**) retinas. White arrows indicate RxRg+ve cones in the outer part of the NBL. NBL: neuroblastic layer. GCL: ganglion cell layer. Scale bars: 30μm. Scale bars of insets: 10μm.


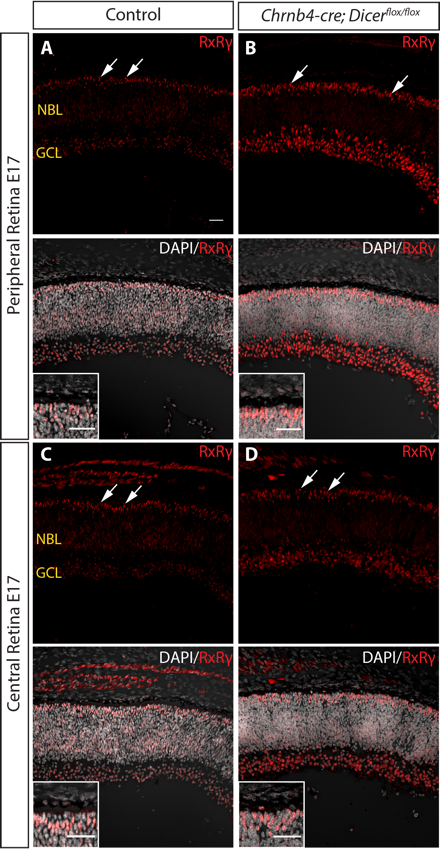


**Supplementary Figure 9**

Single channels shown for data presented in Figure 4A & B. CHX10-expressing bipolar cells in Dicer CKO mice at P21. (**A-D**) Immunohistochemistry for bipolar cell marker CHX10 in peripheral (**A-B**) and central (**C-D**) retinas. White arrows indicate CHX10+ve bipolar cells. Scale bars: 30μm. Scale bars of insets: 10μm.


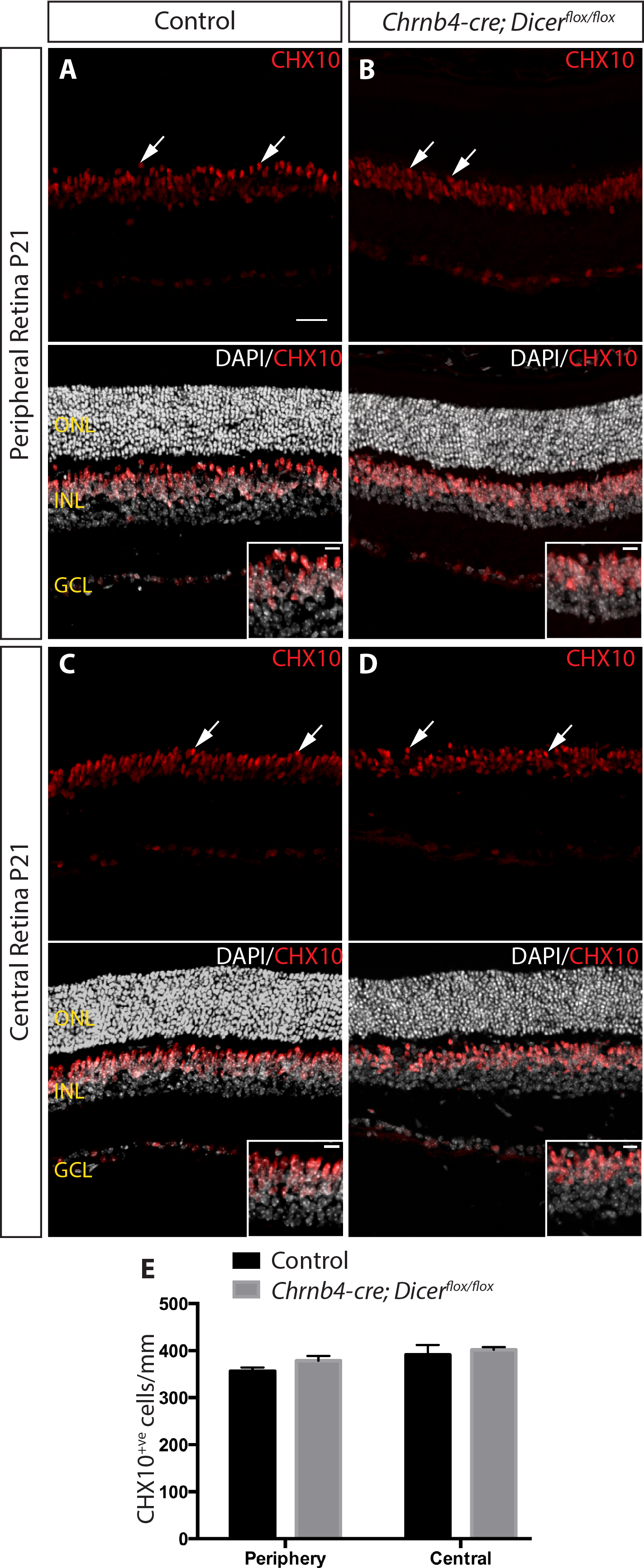


**Supplementary Figure 10**

Single channels shown for data presented in Figure 4H & I. BRN3A-expressing ganglion cells in Dicer CKO mice at P21. (**A-D**) Immunohistochemistry for ganglion cell marker BRN3A in peripheral (**A-B**) and central (**C-D**) retinas. White arrows indicate BRN3A+ve ganglion cells. Scale bars: 30μm. Scale bars of insets: 10μm.


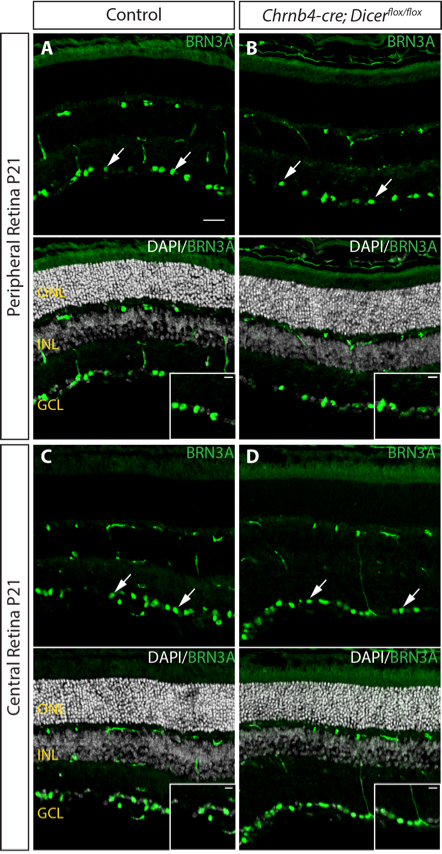


**Supplementary Figure 11**

Single channels shown for data presented in Figure 4K,L. OC1-expressing horizontal cells in Dicer CKO mice at P21. (**A-D**) Immunohistochemistry for horizontal cell marker OC1 in peripheral (A-B) and central (**C-D**) retinas (white arrows). Scale bars: 30μm. Scale bars of insets: 10μm.


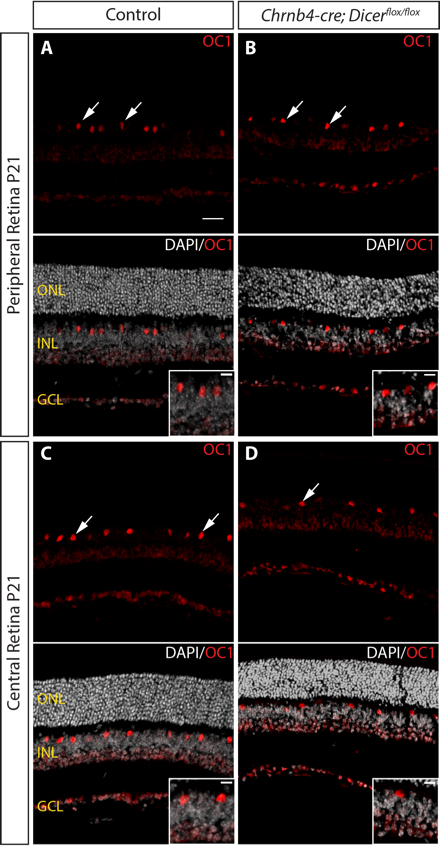


**Supplementary Table 1. List of significantly downregulated genes in Dicer CKO retinas.** 72 genes were significantly downregulated in Dicer CKO P21 whole retinas. Significance was determine by q-value (adjusted p-value) <0.05. q-values were obtained by correcting p-values for multiple testing using the Benjamini-Hochberg False Discovery Rate (FDR).

**Supplementary Table 2. List of significantly upregulated genes in Dicer CKO retinas.** 39 genes were significantly upregulated in Dicer CKO P21 whole retinas. Significance was determine by q-value (adjusted p-value) <0.05. q-values were obtained by correcting p-values for multiple testing using the Benjamini-Hochberg False Discovery Rate (FDR).

**Supplementary Table 3. Top GO terms for biological process.** Gene ontology enrichment analysis of the 111 differentially expressed genes fir biological processes.

**Supplementary Table 4. Top GO terms for molecular function.** Gene ontology enrichment analysis of the 111 differentially expressed genes for molecular function.

**Supplementary Table 5.** Gene Set Enrichment analysis of RNAseq data comparing Dicer CKO and control neural retina at postnatal day 21.

Supplementary
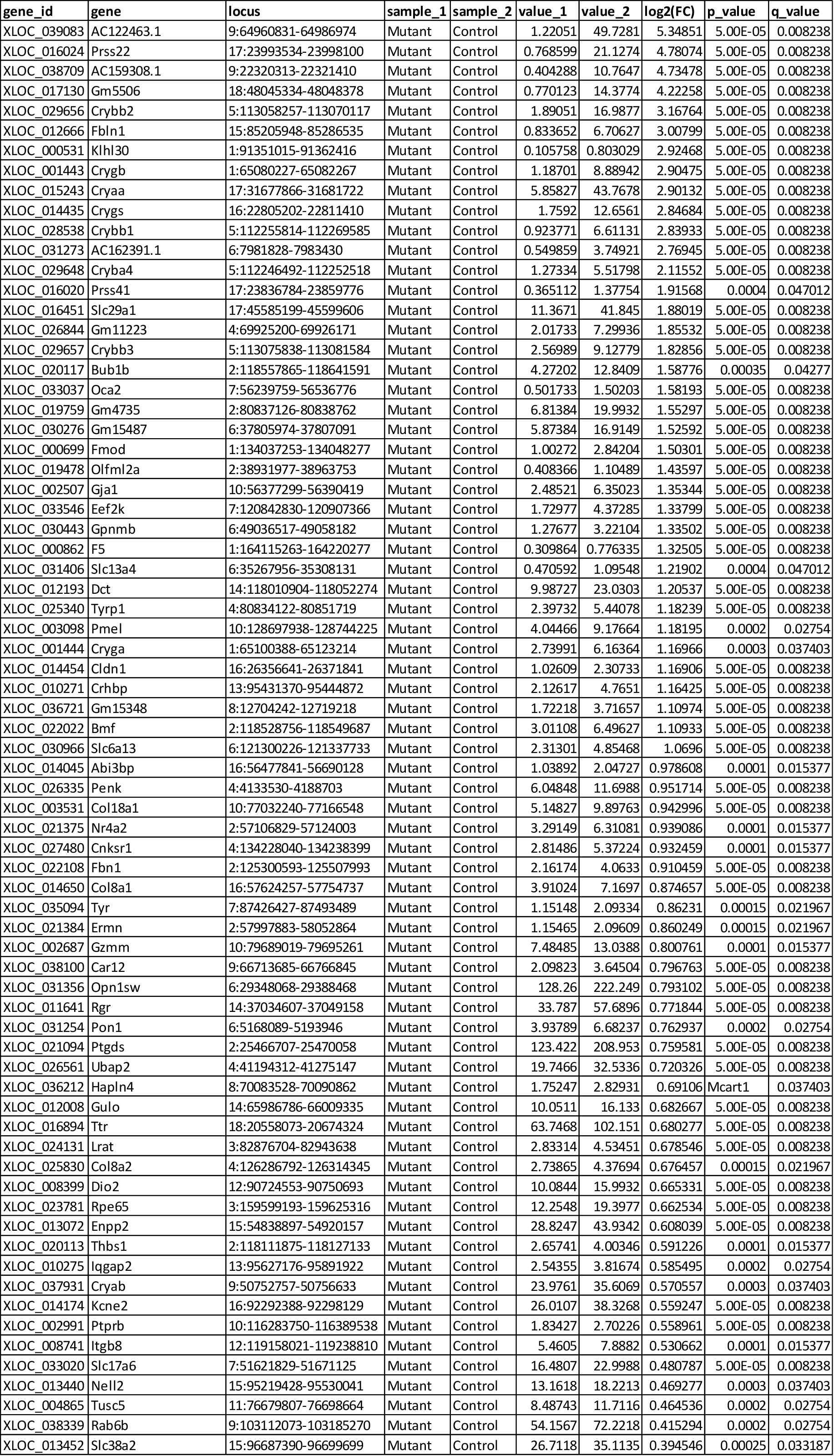
Table 1

Supplementary
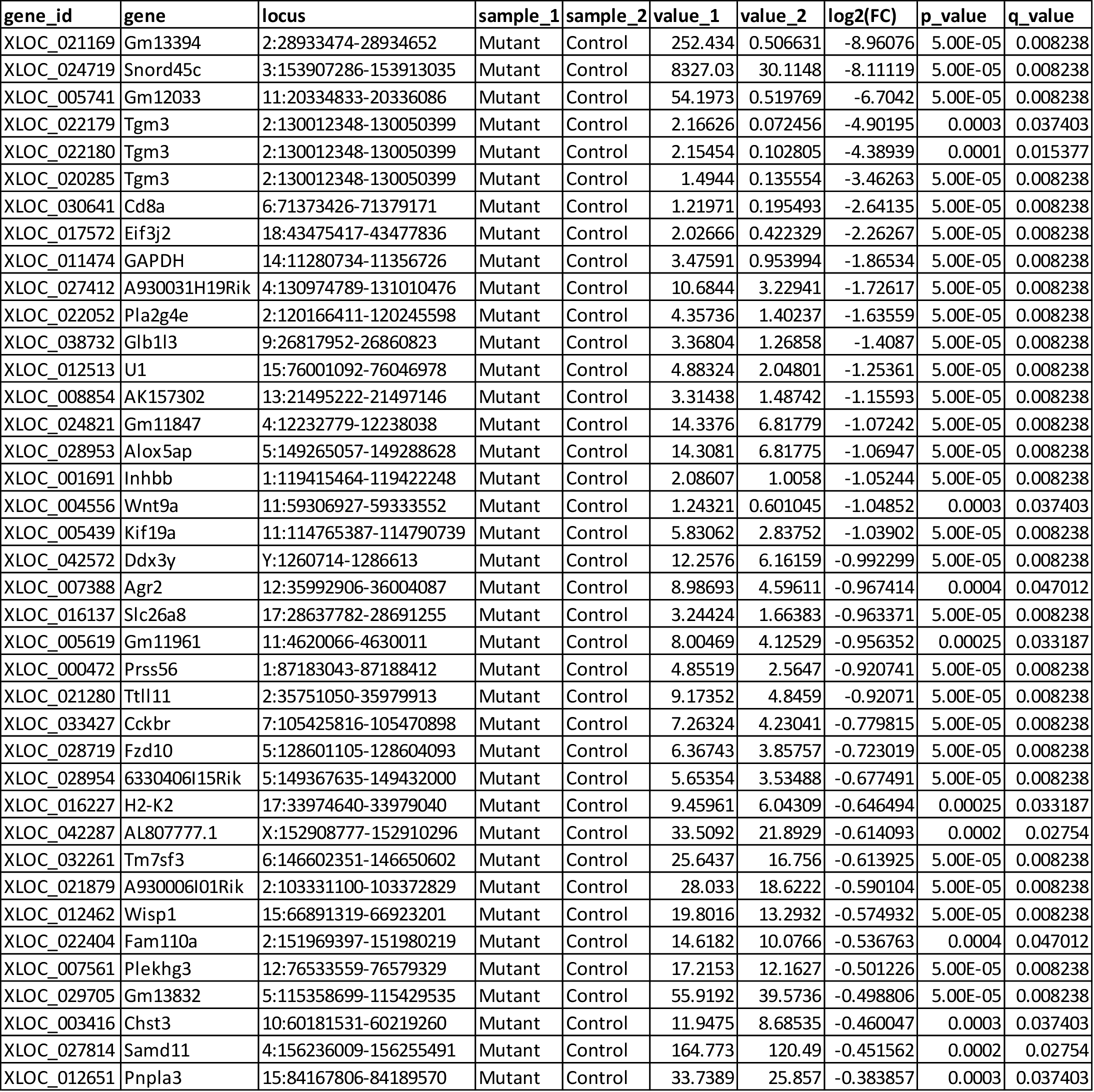
Table 2

Supplementary
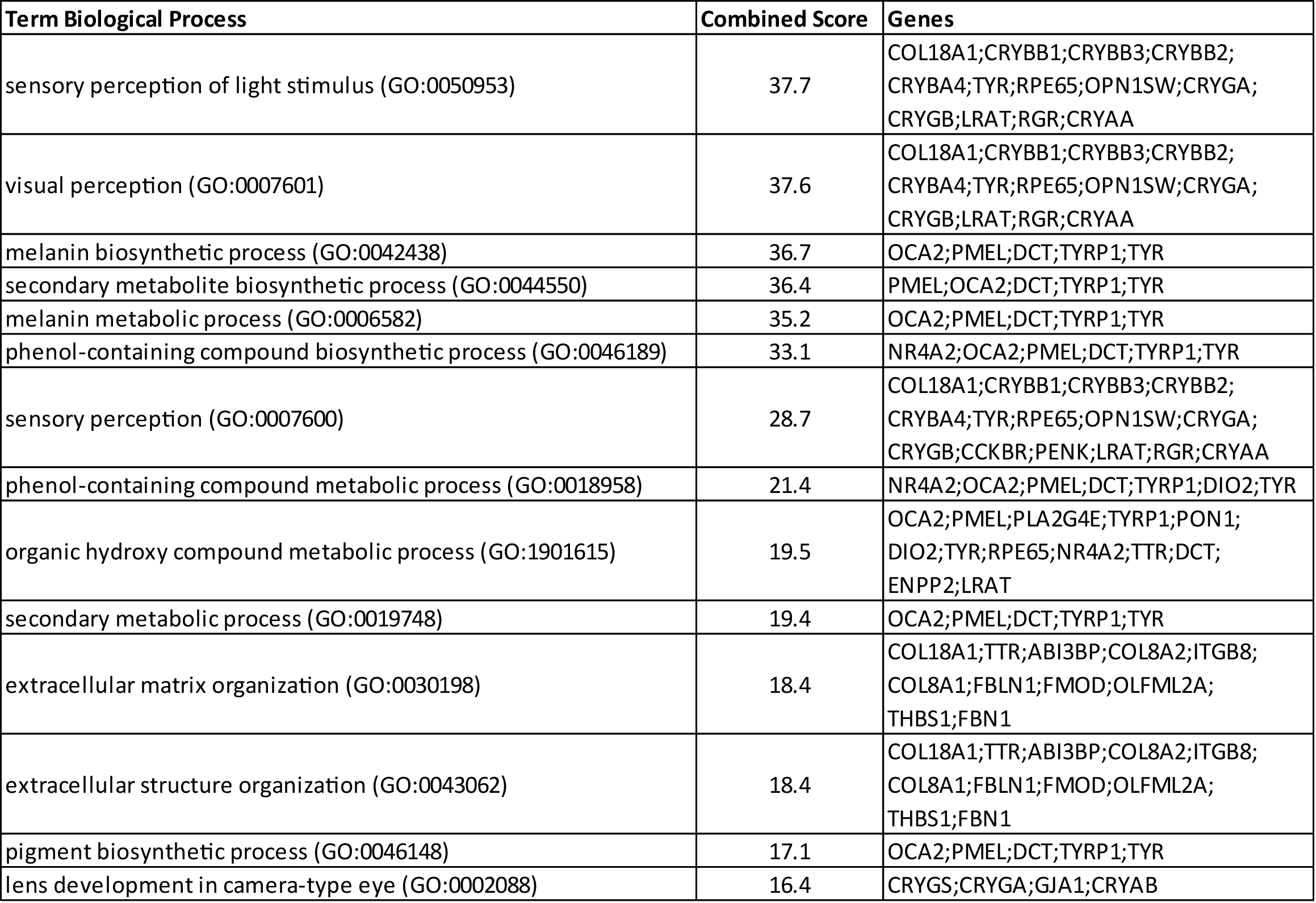
Table 3

Supplementary Table 4


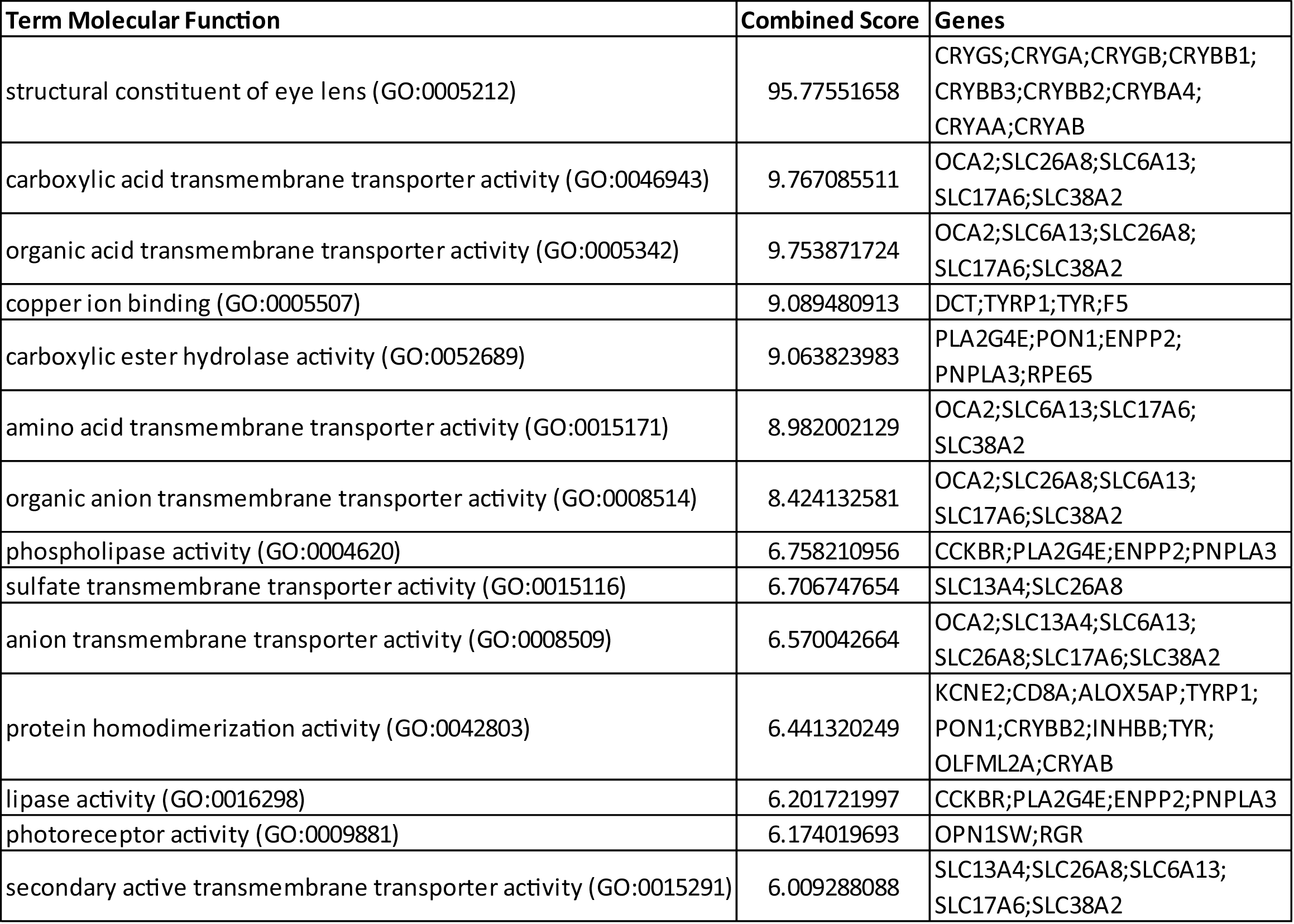


**Supplementary Table 5**: Gene Set Enrichment analysis of RNAseq data comparing Dicer CKO and control neural retina at postnatal day 21.

| Hallmark Gene Set Name | NES (Normalized Enrichment Score ) | FDR q-val |
| --- | --- | --- |
| **POSITIVELY CORRELATED WITH DICER CKO** |  |  |
| HALLMARK_EPITHELIAL_MESENCHYMAL_TRANSITION | 1.798 | 0.0137 |
| HALLMARK_TGF_BETA_SIGNALING | 1.588 | 0.0679 |
| HALLMARK_ANGIOGENESIS | 1.575 | 0.0544 |
| HALLMARK_WNT_BETA_CATENIN_SIGNALING | 1.574 | 0.0411 |
| HALLMARK_APOPTOSIS | 1.456 | 0.113 |
| HALLMARK_COAGULATION | 1.403 | 0.1606 |
| HALLMARK_APICAL_SURFACE | 1.36 | 0.1977 |
| **NEGATIVELY CORRELATED WITH DICER CKO** |  |  |
| HALLMARK_ALLOGRAFT_REJECTION | -1.605 | 0.0627 |


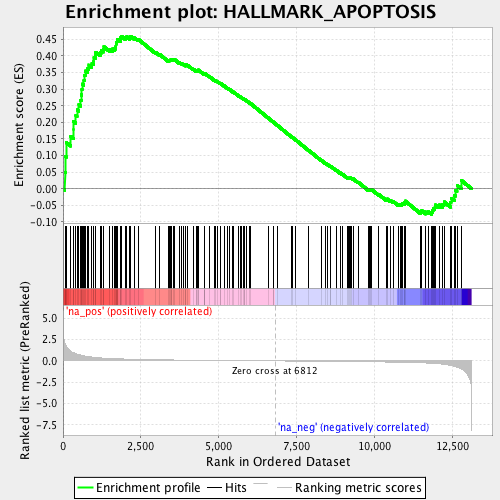


Genes from the Hallmark Apoptosis Gene Set that showed core enrichment in Dicer CKO were:

*CASP4, LUM, GSTM1, BMF, CASP8, BMP2, AIFM3, BGN, CCND2, GCH1, TSPO, PLCB2, BIRC3, DCN, TNFSF10, HSPB1, TIMP3, GSN, CD14, GPX3, CASP1, HMOX1, TGFBR3, TNFRSF12A, TAP1, HGF, LEF1, BID, NEFH, IER3, FAS, F2, CDK2, PDGFRB, TIMP2, F2R, APP, CDKN1A, WEE1, AVPR1A, DPYD, CD38.*

These positively correlated genes were upregulated in the Dicer CKO retina.

Enrichment score (ES), on the Y-axis, is plotted against the list of the genes ranked by Log2 Fold Change, on the X-axis. Gene Set Enrichment Analysis (GSEA) used the 50 Hallmark curated gene sets from the Molecular Signature Database^77^ and a False Discovery rate threshold of FDR<0.25 as recommended^78^
